# The chaperone PrsA2 regulates the secretion, stability, and folding of listeriolysin O during *Listeria monocytogenes* infection

**DOI:** 10.1128/mbio.00743-24

**Published:** 2024-05-29

**Authors:** Charles Agbavor, Adriana Zimnicka, Allison Kumar, Jada L. George, Madeline Torres, Gerd Prehna, Francis Alonzo, Jacob D. Durrant, Nancy E. Freitag, Laty A. Cahoon

**Affiliations:** 1 Department of Biological Sciences, University of Pittsburgh, Pittsburgh, Pennsylvania, USA; 2 Department of Pharmaceutical Sciences, University of Illinois Chicago, Chicago, Illinois, USA; 3 Department of Microbiology, University of Manitoba, Winnipeg, Manitoba, Canada; 4 Department of Microbiology and Immunology, University of Illinois Chicago, Chicago, Illinois, USA; Universite de Geneve, Geneva, Switzerland

**Keywords:** *Listeria monocytogenes*, PrsA2, LLO, chaperone, pore-forming toxin, PPIase

## Abstract

Pathogenic bacteria rely on secreted virulence factors to cause disease in susceptible hosts. However, in Gram-positive bacteria, the mechanisms underlying secreted protein activation and regulation post-membrane translocation remain largely unknown. Using proteomics, we identified several proteins that are dependent on the secreted chaperone PrsA2. We followed with phenotypic, biochemical, and biophysical assays and computational analyses to examine the regulation of a detected key secreted virulence factor, listeriolysin O (LLO), and its interaction with PrsA2 from the bacterial pathogen *Listeria monocytogenes* (*Lm*). Critical to *Lm* virulence is internalization by host cells and the subsequent action of the cholesterol-dependent pore-forming toxin, LLO, which enables bacterial escape from the host cell phagosome. Since *Lm* is a Gram-positive organism, the space between the cell membrane and wall is solvent exposed. Therefore, we hypothesized that the drop from neutral to acidic pH as the pathogen is internalized into a phagosome is critical to regulating the interaction of PrsA2 with LLO. Here, we demonstrate that PrsA2 directly interacts with LLO in a pH-dependent manner. We show that PrsA2 protects and sequesters LLO under neutral pH conditions where LLO can be observed to aggregate. In addition, we identify molecular features of PrsA2 that are required for interaction and ultimately the folding and activity of LLO. Moreover, protein-complex modeling suggests that PrsA2 interacts with LLO via its cholesterol-binding domain. These findings highlight a mechanism by which a Gram-positive secretion chaperone regulates the secretion, stability, and folding of a pore-forming toxin under conditions relevant to host cell infection.

**IMPORTANCE:**

*Lm* is a ubiquitous food-borne pathogen that can cause severe disease to vulnerable populations. During infection, *Lm* relies on a wide repertoire of secreted virulence factors including the LLO that enables the bacterium to invade the host and spread from cell to cell. After membrane translocation, secreted factors must become active in the challenging bacterial cell membrane-wall interface. However, the mechanisms required for secreted protein folding and function are largely unknown. *Lm* encodes a chaperone, PrsA2, that is critical for the activity of secreted factors. Here, we show that PrsA2 directly associates and protects the major *Lm* virulence factor, LLO, under conditions corresponding to the host cytosol, where LLO undergoes irreversible denaturation. Additionally, we identify molecular features of PrsA2 that enable its interaction with LLO. Together, our results suggest that *Lm* and perhaps other Gram-positive bacteria utilize secreted chaperones to regulate the activity of pore-forming toxins during infection.

## INTRODUCTION

Cells depend on molecular chaperones to maintain a functional proteome (1). Chaperones recognize and bind to nascent polypeptide chains or partially folded intermediates to prevent protein aggregation and misfolding (2). During infection, pathogenic bacteria rely on dedicated chaperones critical for the folding and activity of secreted virulence factors at the frontline of host-pathogen interactions (3–5). While a large amount of work has been aimed at identifying secreted virulence factors in Gram-positive bacteria (3, 6, 7), the crucial question still remains as to how secreted virulence factors are folded and regulated post-membrane translocation.

*Listeria monocytogenes* (*Lm*) is a Gram-positive bacterium that typically resides in the environment but also retains the ability to become a facultative intracellular pathogen (8). Upon human ingestion of contaminated food, the bacterium gains entry into the gastrointestinal tract. In vulnerable populations, *Lm* crosses the intestinal barrier, enters the bloodstream, and migrates to distal organs, where it can replicate to high numbers (9–11). Within the infected host, a master transcriptional regulator known as PrfA becomes activated and upregulates the expression of several secreted virulence factors that include the internalins (InlA and InlB) for cell uptake, phospholipases (PI-PLC and PC-PLC), the cholesterol-dependent pore-forming toxin listeriolysin O (LLO) for phagosome escape into the host cytosol, and the bacterial surface protein ActA that directs host actin polymerization to facilitate bacterial cell-to-cell spread (9, 10, 12). Mutant alleles of *prfA* have been identified that result in constitutive activation of PrfA (prfA* alleles), and strains containing prfA* secrete elevated levels of numerous virulence factors and are hyper-virulent (13, 14). The prfA* strain hyper-expressed virulence factors include LLO and the secreted peptidyl prolyl isomerase (PPIase) chaperone PrsA2. Supporting the critical importance of Gram-positive secretion chaperones, the deletion of *prsA2* even in the presence of prfA* causes a significant reduction in bacterial fitness in broth culture and for bacteria within the cytosol of infected cells, presumably due to the accumulation of misfolded secreted proteins at the cell surface (15). Moreover, in the presence of the wild-type *prfA* allele, the deletion of *prsA2* results in a reduction in secreted LLO-dependent hemolytic activity, and Δ*prsA2 Lm* spp. are highly attenuated for virulence in mouse models of infection (15–19). Interestingly, PrsA2 and LLO are translationally coupled, which may result in coordinated protein secretion across the bacterial membrane and in the PrsA2 catalyzed folding of LLO, although this hypothesis remains to be directly tested (20).

PrsA2 is a secreted 32.7-kDa parvulin-type PPIase that is lipid modified by diacylglycerol transferases and localized to the extracellular side of the bacterial cell membrane (21). PrsA2 belongs to a family of PrsA and PrsA-like chaperones that are widely found in Gram-positive bacteria and are thought to be critical for the folding and maturity of secreted proteins and virulence factors (22). With an estimated number of 20,000 molecules per cell, PrsA is likely the most abundant chaperone on the bacterial membrane surface (23). Given that the deletion of *prsA2* results in the accumulation and aggregation of secreted virulence factors, including LLO, at the bacterial membrane interface (15, 16, 18, 24), we set out to examine how the PrsA2 chaperone potentially regulates the stability, folding, and release of LLO to better understand PrsA2 contributions to virulence factor secretion and activity.

Here we provide the first evidence of a direct functional interaction between a PrsA family member (*Lm* PrsA2) and its client (LLO). PrsA2 was found to directly interact with folded/partially folded LLO at a pH of 6.5 and was also shown to protect LLO from aggregation and loss of activity at neutral pH at 37°C. Additionally, PrsA2 binds to LLO with high affinity at neutral pH but binds poorly to LLO under acidic conditions (pH 5.5), suggesting that a drop in pH facilitates the release of LLO from PrsA2, which then helps promote LLO-dependent disruption of the host cell phagosomal membrane. Using protein-complex modeling, we show that the cholesterol-binding domain of LLO may interact with both the PrsA2 foldase domain cavity and the dynamic PPIase domain arms. Taken together, our results support a model in which PrsA2 binds, folds, and sequesters LLO in an active form until LLO release is triggered by the drop in pH encountered by bacteria within host cell phagosomes. Release of primed LLO then allows LLO to bind cholesterol in eukaryotic membranes and form pores required for phagosome disruption and bacterial escape.

## RESULTS

### Identifying the spectrum of PrsA2-dependent secreted proteins in *Lm*

**Fig 1:**
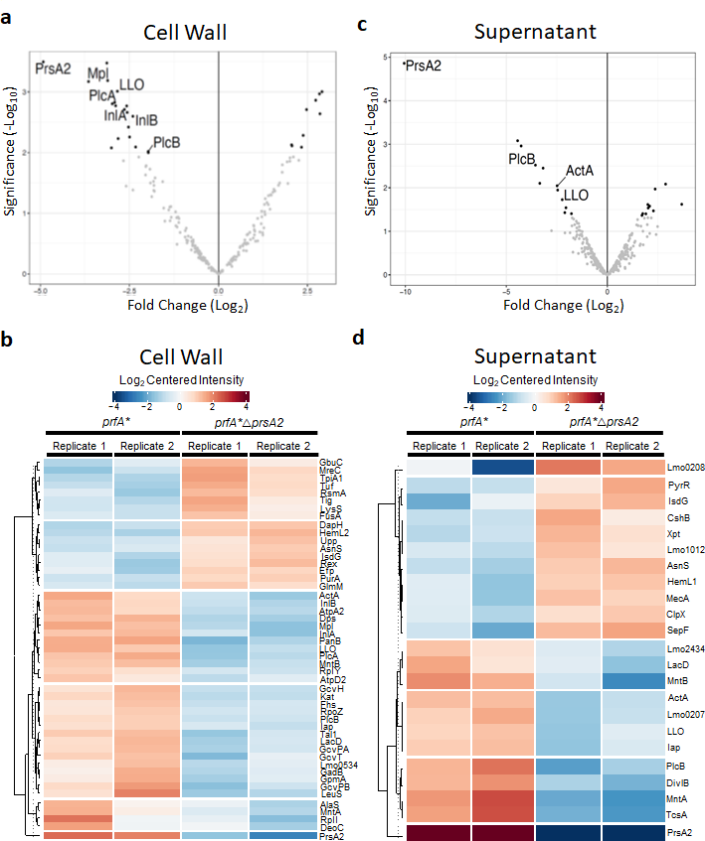
*Lm* PrsA2 is required for the secretion and release of LLO and key virulence factors when PrfA is activated. Volcano plot and heatmap analysis of TMT-MS results demonstrating the fold change of proteins in the Lm prfA* vs Lm prfA*Δ*prsA2* mutant, separately considering the cell wall (**a and b**) and supernatant (**c and d**) protein fractions. Proteomic analysis was performed on the cell wall (**a and b**) or supernatant (**c and d**) fractions of each mutant. Volcano plots represent differentially regulated proteins that are increased or decreased with a 70% confidence interval and log_2_ fold change of >1, prfA*Δ*prsA2* mutant vs prfA* strain. Significant proteins identified in the TMT-MS data are shown as deep black dots, and proteins within the *prfA* virulence regulon and PrsA2 are labeled. All other proteins identified in the TMT analysis are shown as gray dots. Two replicates were performed for each condition, labeled replicates 1 and 2, respectively. Volcano plots and heatmaps were rendered using R software (v.4.3.0), DEP package (25).

To obtain a more complete picture of the diversity of secreted proteins that rely on PrsA2 for folding and/or activity under infection-simulated conditions (prfA* strains), we used tandem mass tagged mass spectrometry (TMT-MS) to analyze bacterial fractions of secreted cell wall proteins and supernatants from broth cultures of Lm prfA* and prfA*Δ*prsA2* cells. Of the total cell wall proteins identified, 21.7% significantly differed in abundance between prfA* and prfA* Δ*prsA2* strains (Fig. 1a and b; Table S1). Further grouping based on functional clustering using the Kyoto Encyclopedia of Genes and Genomes pathway analysis demonstrated that 77.5% of these proteins are predicted to be important for roles associated with *Lm* physiology, while 22.4% are associated with bacterial intracellular growth and virulence (Table S1). Similarly, the differential abundance of supernatant proteins revealed a distribution such that 69.5% and 30.4% of the identified proteins are associated with bacterial physiology and virulence, respectively (Fig. 1c and d; Table S2). Of the 49 PrsA2-dependent genes that were significant in the cell wall fractions, 17 were decreased and 32 were increased in abundance (Fig. 1b). In contrast, 33 proteins were PrsA2 dependent in supernatant cell fractions. Eleven of these proteins were downregulated, while 22 were upregulated (Fig. 1d). By comparison, the *prfA* regulon virulence factors InlA, InlB, and PlcA were identified as less abundant in the absence of *prsA2*, in addition to the previously identified LLO, ActA, Mpl, and PlcB (13, 15).

Unique PrsA2-dependent secreted factors that were significantly different in cell wall and supernatant fractions include proteins important for cell shape (MreC and DivlB), protein biosynthesis (GcvPB), transport (MntA and MntB), and immune evasion and response (Iap and TcsA) (Tables S1 and S2). Multiple cytoplasmic proteins were also identified. However, this was not unexpected, given that 70% of these proteins were previously identified in *Lm* wild-type or prfA* supernatants derived from cells with no apparent membrane alterations or observed cell lysis, or death (6, 7, 15, 26) (Fig. 1a through d; Tables S1 and S2). These results further support a role for PrsA2 in the secretion of a variety of *Lm* proteins, including virulence factors required for *Lm* pathogenesis.

### PrsA2 directly interacts with LLO

The reduction in secreted LLO protein (Fig. 1a through d) and associated hemolytic activity in the absence of *prsA2* (16) suggests that PrsA2 is required for the folding and stability of LLO. To determine whether PrsA2 physically interacts with LLO, we used co-immunoprecipitation of the two proteins from bacterial supernatants as an initial approach. We found that antibodies directed against LLO also brought down PrsA2 from concentrated Lm prfA* and wild-type strain supernatants (Fig. S1a and b), indicating a possible interaction between PrsA2 and LLO. To further explore this potential PrsA2-LLO interaction, we first examined the particle size distribution of recombinant LLO at several different pH values. LLO has previously been shown to aggregate at pH 7.4 at 37°C while remaining soluble at lower pH levels (12). We therefore examined LLO particle size at pH values approximating the host phagosome (pH 5.5), cytosol (pH 7.4), as well as an intermediate value (pH 6.5) at 37°C using dynamic light scattering, with the rationale that chaperones typically interact with unfolded or loosely folded proteins (2). The particle size distribution of LLO at pH values of 5.5 and 6.5 demonstrated that LLO formed monomers as well as oligomers at these pH levels and temperature and that the LLO protein molecule was stable over the time course of our experiments (Fig. S2a through d; Table S3). However, at a pH value of 7.4 and at 37°C, the LLO intensity peaks revealed LLO molecules in oligomeric and/or aggregated states consistent with previous reports that LLO forms insoluble aggregates under these conditions (Fig. S2a through d; Table S3) (12). Interestingly, we found that a subpopulation of LLO molecules appeared partially folded at pH of 6.5 with an average hydrodynamic radius of 26.41 nm compared to LLO at a pH value of 5.5, which had an average hydrodynamic radius of 17.56 nm (Fig. S2a, b, and d; Table S3).

**Fig 2:**
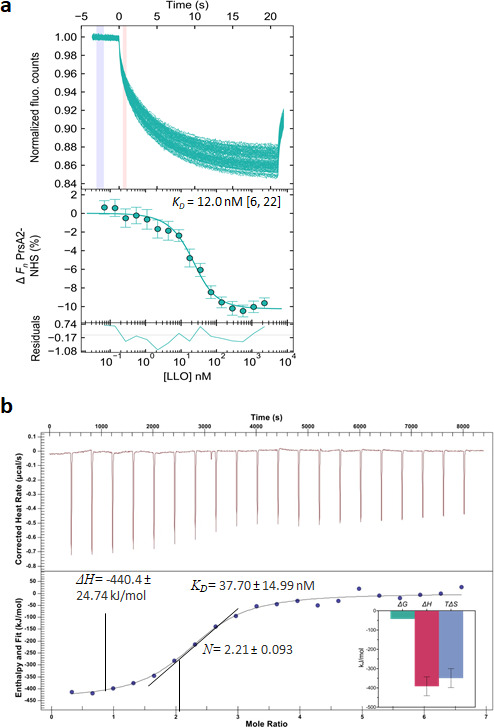
*Lm* PrsA2 physically interacts with folded/partially folded LLO. (a) Thermophoresis of PrsA2 labeled with the NHS dye titrated against unlabeled LLO target. Results were analyzed using PALMIST (27), and the figure was rendered using GUSSI (28) (v.1.2.0). The top panel shows the thermophoretic time traces of four experiments, with blue and pink areas depicting time spans used to obtain the fluorescence cold (*F*_c_) and hot (*F*_h_) regions, respectively. Time traces are normalized to a starting value of 1.0. The middle panel shows the binding curve with baseline fluorescence subtracted, and the error bars represent the standard deviation. The line of best fit was determined using the 1:1 binding model with 95% confidence using error surface projection as published previously (27, 28) (see Materials and Methods for details). The bottom panel represents the residuals between the fit and the data. (b) ITC thermogram of PrsA2 titrated against LLO at pH 6.5, 25°C. Recombinant PrsA2 at 19.0 µM was slowly titrated into 0.4 µM LLO. The heat released during binding was determined by integrating the injection peaks normalized using PrsA2 injected into buffer alone. The titration curve was fitted using an independent model, and the resulting thermodynamic parameters are *N* ~ 2 monomers of PrsA2 to 1 monomer of LLO (2.2:1.0), *K*_D_ = 37.79 ± 14.99 nM, Δ*H* = −440.4 ± 24.74 kJ/mol, *K*_*a*_ = 2.653E7M^(−1)^, Δ*G* = −42.37 kJ/mol, and Δ*S* = −1,335 J/mol·K, *−T*Δ*S* = 398.0 kJ/mol. The inside panel shows the Δ*G,* Δ*H,* and –*T*Δ*S*. Errors were determined from the independent fit of representative isotherms. All experiments were performed at least three independent times.

Since LLO molecules appeared as a folded and partially folded mixture at a pH value of 6.5, we examined whether PrsA2 could directly interact with LLO at pH 6.5. We used several biophysical approaches starting with microscale thermophoresis (MST), which measures the interaction between biomolecules using fluorescent dyes. Recombinant PrsA2 was labeled with the amine reactive dye, N-hydroxysuccinimide (NHS) and titrated against unlabeled LLO at a pH value of of 6.5. We determined that PrsA2 interacts with this mixture of folded and partially folded LLO with a dissociation constant (*K*_*D*_) of 12.0 (6–22) nM (Fig. 2a; Table 1). As fluorescent dyes can interfere with biomolecular interactions, we next used isothermal titration calorimetry (ITC) to measure the binding interaction between unlabeled purified PrsA2 and LLO at a pH value of of 6.5 at 25°C using appropriate controls (Fig. S3a). The thermodynamic parameters show a *K*_*D*_ of 37.70 ± 14.99 nM and stoichiometry of 2:1, suggesting that one PrsA2 dimer interacts with one LLO monomer (Fig. 2b; Table 1). Additionally, the thermodynamic profile of the association between PrsA2 and folded/partially folded LLO (Fig. 2b, bar inset) predicts a spontaneous reaction, suggesting that the observed binding interaction likely occurs under physiological conditions. Both the MST and ITC results are at the same order of magnitude, indicating that the two biophysical methods are complementary (Fig. 2a and b; Table 1).

**TABLE 1:** Thermophoretic and thermodynamic parameters of the interaction between PrsA2 and LLO

Parameters	MST	ITC^*b*^					
	*K*_*D*_ (nM)^*a*^	*K*_*D*_ (nM)	*n*	△*H* (kJ/mol)	△*G* (kJ/mol)	−*T*△*S* (kJ/mol)	△*S* (J/mol·K)
PrsA2-LLO, pH 5.5							
25^o^C	150.0 (70, 320)	201.0 ± 61.22	1.962 ± 0.059	−33.16 ± 1.496	−38.23	−5.06	16.98
37^o^C	190.0 (70, 470)	330.30 ± 127.1	1.948 ± 0.063	−22.97 ± 1.132	−38.43	−15.52	50.03
PrsA2-LLO, pH 6.5							
25^o^C	12.0 (6, 22)	37.70 ± 14.99	2.21 ± 0.093	−440.4 ± 24.74	−42.37	398.0	−1335
37^o^C	28.0 (18, 40)	88.32 ± 31.11	1.729 ± 0.058	−129.1 ± 6.542	−41.88	300.4	−281.1
PrsA2-LLO, pH 7.4							
25^o^C	23.0 (14, 33)	46.10 ± 22.18	2.11 ± 0.046	−90.96 ± 2.98	−43.56	47.40	−152.80
37^o^C	39.0 (21, 66)	67.23 ± 18.56	2.012 ± 0.029	−53.65 ± 1.25	−42.59	11.07	−35.68

^*a*^ Denotes the dissociation constant (*K*_*D*_) from microscale thermophoresis (MST) experiments. *K*_*D*_ was determined using the default 1:1 model with a 95% confidence interval (upper and lower values in parentheses) in PALMIST based on error surface projection.

^*b*^ Denotes the representative ITC thermograms of PrsA2 titrated into purified LLO protein at different pH levels. *K*_*D*_ is the dissociation constant; △*H* is the reaction enthalpy; *n* is the stoichiometry between PrsA2 and LLO; and the Gibbs free energy relation of the interaction between PrsA2 and LLO is shown*.* The error of each parameter was determined from the independent fitting model applied to the ITC data using a 95% confidence interval. All experiments were performed at least three independent times.

To determine whether the binding interaction between PrsA2 and folded/partially folded LLO occurs at the physiological temperature of the human host, we defined the interaction of PrsA2 with LLO at 37°C. As anticipated, PrsA2 bound to folded/partially folded LLO at 37°C with nanomolar affinity as measured by MST and ITC, with *K*_*D*_ values of 28 and 88.32 nM, respectively (Table 1; Fig. S3b and c). The higher affinity of PrsA2 for LLO at 25°C compared to 37°C suggests PrsA2 may tightly regulate the secretion and release of LLO at lower temperatures and in comparison to temperatures found within mammalian host cells, where the release of LLO is anticipated to be important to promote infection. Taken together, these results indicate that PrsA2 physically associates with folded/partially folded LLO.

### PrsA2 binds with poor affinity to LLO at pH 5.5 and 37^o^C

Since PrsA2 is bound to LLO at pH 6.5 at 25°C and 37°C (Table 1), we asked whether PrsA2 interacts with LLO under physiological conditions approximating the phagosome. Using MST, we determined the binding affinity of PrsA2 to LLO at pH 5.5, which corresponds to the approximate pH within the phagosome and tested temperatures of 25°C (outside environment) and 37°C (host cytosol). PrsA2 interacts with LLO at pH 5.5 at 25°C and 37°C with *K*_*D*_ values of 150 and 190 nM, respectively (Table 1; Fig. S3d and e). Similarly, ITC thermodynamic parameters showed that PrsA2 interacts with LLO at pH 5.5 with *K*_*D*_ values of 201 and 330 nM at 25°C and 37°C, respectively (Table 1; Fig. S3f and g). The affinity of PrsA2 to LLO at pH 5.5 suggests that PrsA2 is likely associating and rapidly folding and releasing a large amount of functional LLO necessary for pore formation and breakdown of the phagosomal membrane. Together, these results suggest that PrsA2 associates with LLO under physiological conditions relevant to *Lm* infection within the phagosome.

### PrsA2 binds with high affinity to LLO at neutral pH and 37°C

We next examined potential PrsA2-LLO interactions under conditions relevant to bacteria within the human cell cytosol (pH 7.4 at 37°C). We used MST and ITC to determine the thermophoretic and thermodynamic interactions, respectively, between PrsA2 and LLO at pH 7.4 and compared different temperatures. Interestingly, PrsA2 bound to LLO at pH 7.4 at 25°C and 37°C with nanomolar affinities as measured by MST (Table 1; Fig. S3h and i). Additionally, the stable interaction between PrsA2 and LLO at pH 7.4 and 25°C was confirmed using ITC (Table 1; Fig. S3j). Because LLO aggregates at a pH value of 7.4 and 37°C (12, 29) (Fig. S2c and d; Table S3), the ITC experiment was not feasible at high LLO concentrations due to non-specific binding. However, at low LLO concentrations, we could mitigate background heats from non-specific self-aggregation. This resulted in a PrsA2-LLO *K*_*D*_ of 67.23 nM (Table 1; Fig. S3k), confirming the MST results indicating that PrsA2 associates with LLO at pH 7.4 and 37°C. Remarkably, the strongest enthalpic binding interactions between PrsA2 and LLO were observed at pH 7.4 (in comparison to pH 5.5), which suggests that a large amount of energy is released by the association between PrsA2 and LLO at pH 7.4 vs pH 5.5 (Table 1). Additionally, the thermodynamic profiles of PrsA2-LLO interactions at pH 6.5 and 7.4 are likely dominated by favorable hydrogen bonds, van der Waals packing, and electrostatic interactions but may include a large conformational change and/or exposure of hydrophobic surfaces as seen by the negative △*H* and positive *−T*△*S* values (Table 1). In contrast, the thermodynamic binding profile at a pH value of 5.5 is both enthalpically and entropically favorable, possibly indicating LLO is fully folded before release by PrsA2 (Table 1). Taken together, these data suggest that PrsA2 interacts with LLO at physiological conditions that mimic those found within the host cell cytosol (pH 7.4 and 37°C).

### The PrsA2 foldase, dimerization, and PPIase domains are required for interaction with LLO

To examine which molecular features of PrsA2 are necessary for interaction with LLO, we took advantage of three PrsA2 mutants previously generated: a foldase domain mutant with a single V91T substitution previously shown to be associated with reduced secreted LLO hemolytic activity (PrsA2 V91T) (30), a mutant containing only the foldase domain (PrsA2 N+C) that lacks the entire PPIase domain and is associated with normal secreted hemolytic activity at pH 5 (17), and a mutant defective in dimer formation that is associated with reduced hemolytic activity (PrsA2 monomer/PrsA2^M^) (30). Alonzo et al. have shown previously that strains containing PrsA2 N+C exhibit normal levels of secreted LLO-dependent hemolytic activity at pH 5 (17). Given this, we examined the *prsA2* N+C strain as well as the other *prsA2* mutants for secreted LLO-dependent hemolytic activity at the pH levels for which we have found that PrsA2 interacts with LLO (Fig. 2a and b; Table 1). Interestingly, all mutant strains exhibited reduced hemolytic activity at the three examined pH levels with the exception of the *prsA2* N+C mutant strain at pH 5.5 (Table 2). This mutant stain had hemolytic activity more comparable to wild-type strains (consistent with previous results) (17). The *prsA2* N+C mutant strain exhibited more substantial reductions in hemolytic activity at pH 6.5 and 7.4 (Table 2), suggesting that the PPIase domain is required for full activity at higher pH. Strains containing the *prsA2^M^* mutation exhibited the most significant reductions in LLO hemolytic activity, while *prsA2* V91T strains were somewhat modestly reduced (Table 2). These data indicate that the PrsA2 foldase, dimerization, and PPIase domain are important for the secretion and release of functional LLO protein.

**TABLE 2:** LLO-dependent hemolytic activity of *Lm* PrsA2 and mutants at pH values of 5.5, 6.5, and 7.4 and at 37°C in liquid broth^*a*^

Strain	Hemolytic units (AU)					
	pH 5.5		pH 6.5		pH 7.4	
	Mean	FR	Mean	FR	Mean	FR
Wild type	100 ± 0.0	–	100 ± 0.0	–	100 ± 17.21	–
Δ*prsA2*	45.83 ± 6.45****	0.54	43.75 ± 6.84****	0.56	34.72 ± 8.19***	0.55
Δ*prsA2 + prsA2*	91.66 ± 12.90	0.08	100 ± 0.0	0	100 ± 17.21	0
Δ*prsA2 + prsA2* V91T	66.66 ± 12.90****	0.33	66.66 ± 12.90****	0.33	58.33 ± 9.12*	0.25
Δ*prsA2 + prsA2^M^*	52.08 ± 12.22****	0.47	47.91 ± 5.10****	0.52	55.55 ± 8.60*	0.28
Δ*prsA2 + prsA2* N+C	87.5 ± 13.69*	0.12	58.33 ± 12.90****	0.41	44.44 ± 8.60**	0.42

^*a*^ *Lm* mutant strains were compared to the *Lm* wild-type strain using unpaired, two-tailed Student *t-*test; *P < 0.05, **P < 0.001, ***P < 0.0005, ****P < 0.0001. Data are represented as mean ± standard deviation. FR, fold reduction.

To determine whether the PrsA2 mutants described above also demonstrated a reduced binding affinity for LLO, we first confirmed that the PrsA2 mutant protein structures were not significantly perturbed by the introduced mutations using circular dichroism spectroscopy (Fig. S4). We additionally confirmed that the PrsA2 dimer mutant (PrsA2^M^) was defective for dimer formation using a chemical crosslinker [bis-(sulfosuccinimidyl) substrate (BS3) crosslinker] prior to SDS-PAGE and Western analysis and showed that the PrsA2^M^ protein exhibited reduced dimer formation (with altered/defective migration) in comparison to the wild-type PrsA2 (Fig. S5). We then determined the binding affinity of PrsA2 mutants for LLO using MST and ITC. PrsA2 V91T, PrsA2 N+C, and PrsA2^M^ mutants were observed to bind to LLO with *K*_*D*_ values of 2.8, 330, and 190 nM, respectively, by MST, and these results were confirmed by ITC (Table 3; Fig. S6a through f). Surprisingly, the V91T mutation within the hydrophobic interface of the PrsA2 foldase domain exhibited a higher binding affinity for LLO in comparison to the PrsA2 wild-type protein, an observation that is perhaps due to changes at the hydrophobic interface of PrsA2. The reduced affinity of the PrsA2 N+C as well as the PrsA2^M^ mutants to LLO suggests that the PrsA2 PPIase domain and PrsA2 dimerization are structurally important for PrsA2 binding to LLO (Table 3). The reduced affinity of the PrsA2^M^ mutant for LLO is supported by predicted stoichiometry indicating that a dimer of PrsA2 interacts with LLO (Fig. 2b).

**TABLE 3:** Thermophoretic and thermodynamic parameters of the interaction between PrsA2 mutants and LLO

	MST		ITC^***b***^				
Parameters	***K***_***D***_ (nM)^***a***^	***K***_***D***_ (nM)	*n*	△*H* (kJ/mol)	*S*△*G* (kJ/mol)	−*T*△*S* (kJ/mol)	△*S* (J/mol·K)
PrsA2 V91T-LLO, pH 6.5, 25°C	2.80 (1.2–5.3)	9.64 ± 4.19	2.11 ± 0.015	−17.62 ± 0.27	−45.75	−28.13	94.36
PrsA2^M^-LLO, pH 6.5, 25°C	190.0 (80–490)	206.2 ± 59.08	2.06 ± 0.04	−89.30 ± 2.70	−38.16	51.14	−171.5
PrsA2 N+C-LLO, pH 6.5, 25°C	330.0 (130–860)	356.7 ± 128.2	2.08 ± 0.074	−69.32 ± 3.21	−36.80	32.51	−109.1

^*a*^ Denotes the dissociation constant (*K*_*D*_) from microscale thermophoresis (MST) experiments. *K*_*D*_ was determined using the default 1:1 model with a 95% confidence interval (upper and lower values in parentheses) in PALMIST based on error surface projection (27).

^*b*^ Denotes the representative ITC thermograms of PrsA2 mutants titrated into purified LLO protein at pH 6.5. *K*_*D*_ is the dissociation constant; △*H* is the reaction enthalpy; and *n* is the stoichiometry between PrsA2 and LLO. The error of each parameter was determined from the independent fitting model applied to the ITC data using a 95% confidence interval. All experiments were performed at least three independent times.

### PrsA2 is critical for the protection and folding of LLO

**Fig 3:**
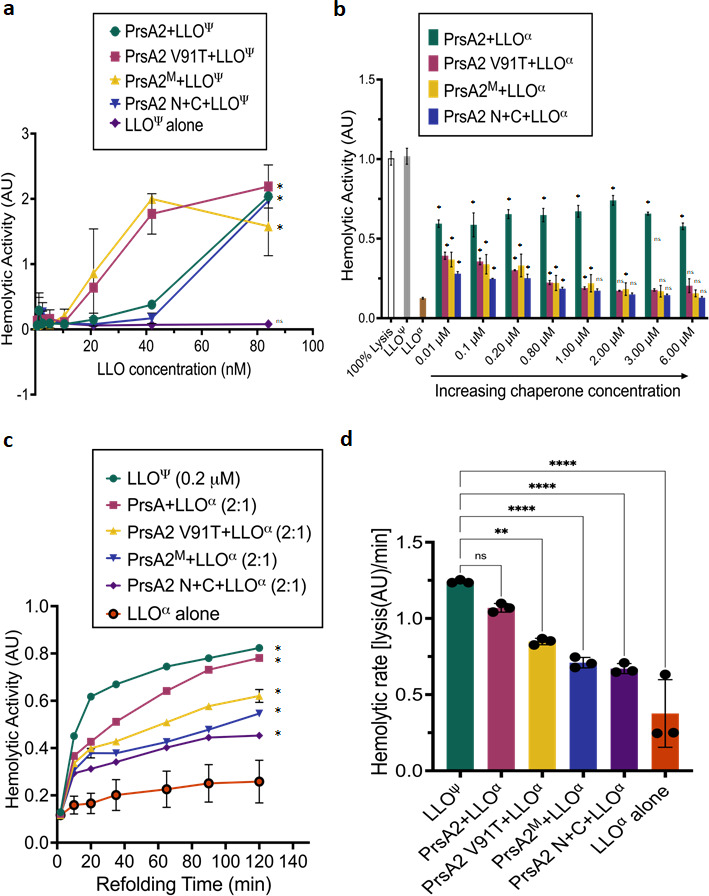
*Lm* PrsA2 protects and folds LLO. (a) PrsA2-LLO protection assay. To allow protein association, recombinant purified wild-type or chaperone mutant was incubated with LLO on ice at pH 7.4 for 15 min at a 30:1 ratio. This incubation was followed by the induction of LLO aggregation at 37°C for 1 hour. Then, chaperone and LLO mixtures were serially diluted with the addition of sheep’s red blood cells (RBCs) and incubated at 30°C for 30 min. After centrifugation, supernatants were assessed for released hemoglobin by measuring absorbance at 415 nm. The resulting activity was determined in hemolytic units measured as the reciprocal of the highest LLO dilution that resulted in 50% lysis of sheep’s RBCs. The final hemolytic activity of LLO alone (80 nM) was compared to PrsA2 and mutants using one-way ANOVA with Dunnett’s multiple comparisons test. *P < 0.05. (b) Chaperone-assisted protein folding assay was used to measure the refolding of urea-denatured LLO by the chaperone PrsA2 at pH of 6.5. For the assay, purified recombinant LLO that was dialyzed at pH 6.5 was used. First, LLO was unfolded in denaturing buffer containing 8-M urea for 45 min (see Materials and Methods for details). After denaturing, the unfolded LLO was diluted 20-fold in assay buffer at pH 6.5. LLO hemolytic activity was measured to determine functional LLO in solution in the presence of PrsA2 or PrsA2 mutants at pH 6.5. Urea-denatured and diluted LLO (0.01 µM) alone, wild-type non-denatured LLO (0.01 µM), and a 100% lysed sample were used as internal controls. The concentration of urea-denatured and diluted LLO (0.01 µM) in the assay was constant, and increasing concentrations of chaperone or mutant proteins were added at 0.01, 0.1, 0.2, 0.8, 1.0, 2.0, 3.0, and 6.0 µM. The hemolytic activity of PrsA2 and mutants was compared to the no-chaperone control using unpaired two-tailed Student *t*-test. *P < 0.05. (c) Time course of chaperone-assisted protein refolding of 8-M urea-denatured LLO at pH 6.5. The hemolytic activity was determined using a 2:1 stoichiometry of PrsA2 or PrsA2 mutant proteins to denatured LLO over 120 min. Wild-type non-denatured LLO and denatured LLO alone were used as controls. The hemolytic activity of all samples was compared to the denatured LLO-alone control using one-way ANOVA with Tukey pairwise comparisons at the 90- and 120-min marks. *P < 0.05. (d) Hemolytic rates (lysis per minute) were determined (**c**) by dividing the hemolytic activity by the refolding time at points where the curves taper at the 1-hour mark. The difference in the hemolytic units/rates was compared using one-way ANOVA with Tukey pairwise comparisons, **P < 0.001, ****P < 0.0001. Ψ indicates fully folded LLO, and ⍺ indicates denatured LLO. Error bars indicate the standard error of the mean. All experiments were performed in triplicate. ANOVA, analysis of variance; ns, not significant.

Our results demonstrate that PrsA2 associates with LLO at neutral pH and at 37°C, at which conditions free LLO has been previously shown to rapidly aggregate and form insoluble precipitates (12, 29). This suggests that PrsA2 functions to stabilize LLO under the aggregation-prone conditions (Tables 1 and 3). This may occur by either binding of PrsA2 to the partially unfolded LLO at neutral pH and 37°C to shield its exposed hydrophobic residues from aggregation or by shielding as well as actively refolding LLO when it becomes unfolded at neutral pH. In both scenarios, a release of LLO is likely to occur when pH is lowered to 5.5 and the binding affinities between LLO and PrsA2 decrease at that pH (from ~67 nM at pH 7.4 to ~330 nM at pH 5.5, Table 1). We developed a protection assay in which purified PrsA2 and LLO proteins were incubated together at pH 7.4 and 37°C at a 30:1 molar ratio for 1 hour. The temperature and pH were then lowered to pH 5.5 and 30°C, and the LLO-dependent hemolytic activity was assessed. We observed that the activity of LLO alone was completely abolished after subjecting it to aggregation-prone conditions (Fig. 3a). In contrast, LLO that was incubated with PrsA2 under aggregation-prone conditions was subsequently capable of significant hemolytic activity, at LLO concentrations above 40 nM.

To determine the critical domains of PrsA2 that may shield and/or refold LLO during the aggregation-prone conditions, we utilized three PrsA2 mutants listed in Table 2. Incubation of LLO at pH 7.4 with either PrsA2 V91T (replacement of hydrophobic valine 91 with polar threonine at the bottom of the foldase domain) or the PrsA2^M^ (no dimerization mutant, no foldase domain formed) resulted subsequently in ~2-fold higher hemolytic LLO activities at pH 5.5, compared to the wild-type PrsA2 protein or PrsA2 N+C mutant at pH 5.5 (Fig. 3a). The LLO protected by V91T or PrsA^2M^ mutants displayed robust hemolytic activity at concentrations as low as 20 nM and saturated at 40 nM. In comparison, LLO protected by the wild-type PrsA2 or N+C mutant with the intact foldase domain displayed hemolytic activity only above 40 nM (Fig. 3a). In summary, the activity of LLO at pH 5.5, after the incubation with PrsA2 mutants at aggregation-prone conditions, was as follows: PrsA2 V91T = PrsA2^M^ >> PrsA2 N+C = PrsA2 wild type. Since protection of LLO by PrsA2 V91T mutant with the highest LLO binding affinity (~2.8 nM at pH 5.5) is high and the same as LLO activity after protection by the PrsA^2M^ mutant (binding affinity of ~330 nM at pH 5.5), this suggested that the PrsA2-binding affinities to LLO are not predictive of subsequent LLO activity. Results also indicate that the presence of the intact foldase domain of PrsA2 did not matter for the subsequent LLO activity, and in fact its disruption, as in the case of PrsA2 V91T or PrsA^2M^ mutants, resulted in the higher activity of LLO afterward at pH 5.5. Similarly, removal of PPIase arms (PrsA2 N+C mutant) did not affect subsequent activity of LLO. This suggests that neither PrsA2 PPIase activity, foldase activity, nor PrsA2 dimerization is important for LLO activity post-incubation at aggregation-prone conditions. The protection assay involves multiple steps, including binding of PrsA2 to LLO at pH 7.4 and then perhaps release, or refolding and release of LLO from PrsA2 at pH 5.5, at which point the LLO hemolytic activity is measured. Thus, the results of the hemolytic protection assay are influenced by a combination of the PrsA2-binding affinity as well as refolding and release rates of LLO. There does not seem to be a simple relation between the ability of PrsA2 to protect LLO activity and its foldase activity.

As PrsA2 binds to folded/partially folded LLO (Table 1), we postulated that PrsA2 accelerates the folding kinetics of unfolded LLO. Therefore, we developed a chaperone-assisted protein folding assay that measures the rate of denatured LLO protein folding in the presence of PrsA2. For this assay, purified LLO is denatured with 8 M urea and diluted 20-fold, and PrsA2 is added at increasing concentrations and incubated for 5 min. Next, the hemolytic activity of functional LLO was measured after incubation with sheep’s red blood cells (RBCs) for 45 min (Fig. 3b). As hypothesized, the addition of PrsA2 wild type to denatured LLO resulted in increased LLO-dependent hemolytic activity compared to the no chaperone incubation control (Fig. 3b). Each of the PrsA2 mutants was also capable of refolding LLO; however, all appeared less efficient than wild-type PrsA2. Interestingly, while increasing the concentration of wild-type PrsA2 did not increase or decrease the total amount of active LLO, the ability of the mutants to refold LLO decreased with increasing mutant chaperone concentration (Fig. 3b). To examine the rate of folding over time, the chaperone-assisted protein folding experiment was performed. For this assay, purified LLO was denatured and diluted 20-fold and PrsA2 wild-type protein was added and incubated for 5 min. Then the LLO-dependent hemolytic activity was measured from the time of addition of red blood cells to 140 min. Since we used the measured stoichiometry of 2:1 between PrsA2 and LLO, respectively (Table 1), the results reflect the relative rates at which PrsA2 accelerates the refolding of unfolded LLO (Fig. 3c). In addition, we determined that the PrsA2 V91T, PrsA2^M^, and PrsA2 N+C mutants demonstrate significantly decreased LLO refolding kinetics (Fig. 3b through d). These experiments indicate that optimal refolding of denatured LLO requires PrsA2 dimerization as well as an active PPIase domain.

## DISCUSSION

The ability of *Lm* to invade and cause disease in susceptible humans is dependent on the primary virulence factor, LLO (9). LLO is the only pH-dependent, phagosome-specific cytolysin produced by an intracellular pathogen, *Lm* (31). Several studies have shown how pH and temperature regulate the activity of LLO such that the membrane of the invaded host cells is not damaged by secreted LLO (10, 12, 29, 32–34). Here, we show how *Lm* uses the PrsA2 chaperone to stabilize, fold, and sequester functional LLO at neutral pH and 37°C until a drop in pH occurs, and *Lm* PrsA2 releases LLO.

**Fig 4:**
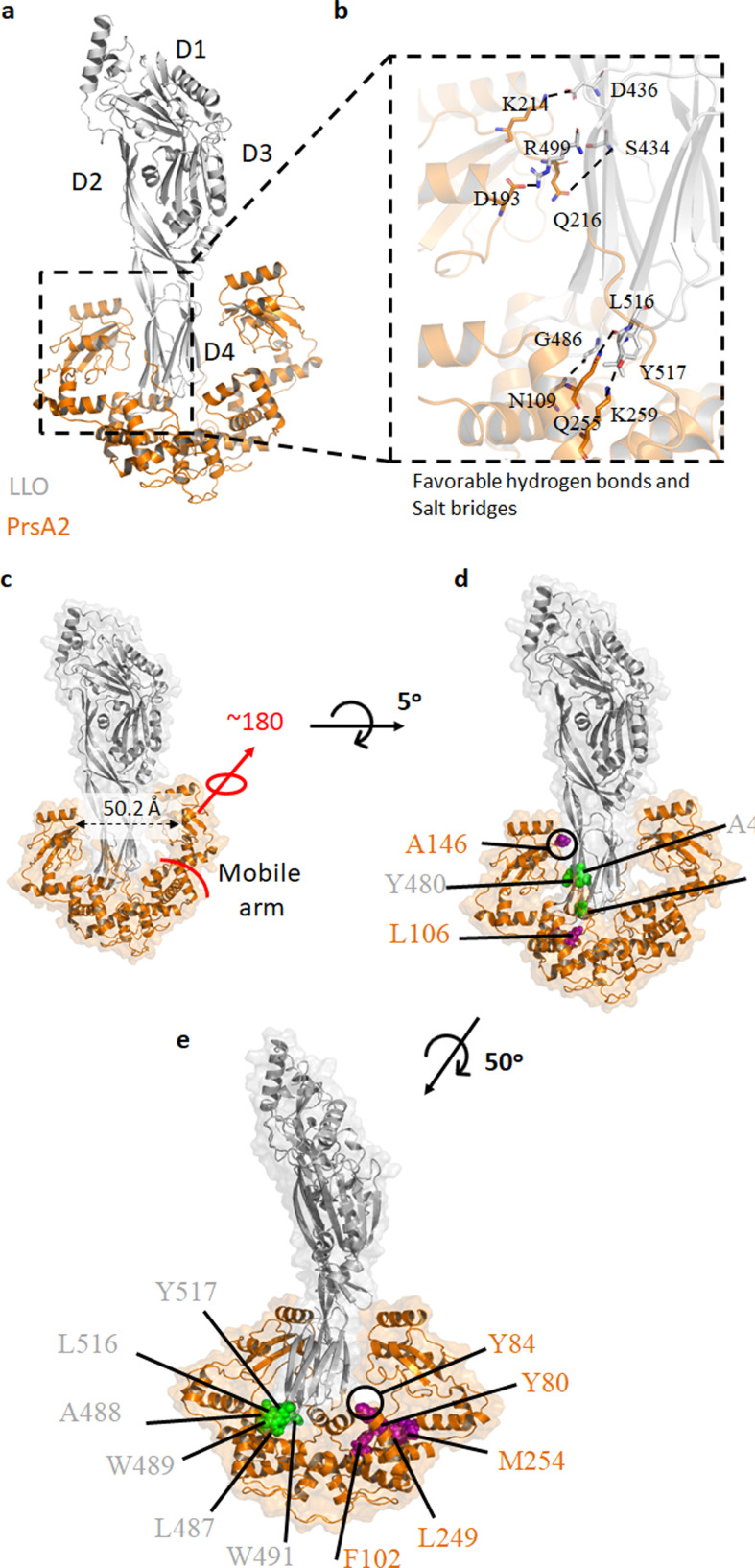
Predicted structure of PrsA2-LLO complex. (a and b) AlphaFold-predicted model of the PrsA2-LLO complex indicating favorable salt bridges and hydrogen bonds between PrsA2 and LLO. The four domains of LLO are labeled as D1–D4. PrsA2 and LLO amino acids are shown as sticks, and key interactions are denoted as black dashed lines. (c–e) Surface representation of the predicted PrsA2-LLO complex. The rotation of the protomeric arms of PrsA2 is approximately 180^o^, and the PrsA2 substrate pocket is approximately 50.2 Å in length. PrsA2 and LLO hydrophobic residues are purple and green, respectively.

Because Gram-positive bacterial pathogens depend on secreted virulence factors such as LLO to invade, replicate, and escape the host immune response (9, 35–37), the folding and maturation of these virulence factors are crucial for bacteria to survive and establish replication niches during infection. Our experiments show that PrsA2 binds to LLO (Tables 1 and 3); therefore, we used AlphaFold-Multimer (38) to predict the structure of the PrsA2-LLO complex (Fig. 4a; Fig. S7).

The predicted complex suggests that PrsA2 binds to the cholesterol-binding domain of LLO with observed favorable hydrogen bonds, salt bridges (Fig. 4a and b), and a large hydrophobic interface containing at least 16 amino acids with hydrophobic side chains (Fig. 4c and e). Several previous studies provide validating evidence for this model. For example, we have previously predicted that tyrosine 80 and 84 of the PrsA2 foldase domain are necessary for client-protein interaction (30), and both residues lie at the AlphaFold-predicted PrsA2-LLO hydrophobic interface. Similarly, we have previously predicted and shown that the PrsA2 valine 91 is important for LLO-dependent activity (30), and the AlphaFold model suggests it forms hydrophobic contacts with LLO W491. Of note, LLO W491 is required for LLO-dependent hemolytic activity, so much so that the W491A substitution reduces that activity by about 95% (39).

The large PrsA2 foldase pocket (50.2 Å) predicted to bind the LLO cholesterol-binding domain (Fig. 4c) suggests that PrsA2 directly regulates LLO pore-forming activity. Further, LLO activity is markedly regulated at both the transcriptional (40) and translational levels. Recent work by Ignatov et al. showed that a short 3′ UTR of *hly* (LLO gene) directly base pairs with the 5′ UTR of *prsA2* mRNA (20). The binding of these transcripts suggests that PrsA2 and LLO are both translationally and secretion coupled to enhance LLO folding and regulation within the host, a mechanism required for efficient replication and spread of *Lm* during infection.

*Lm prsA2* deletion strains are known to have significant reductions in cell-to-cell spread (13, 15, 18) and virulence in mouse models of oral and septicemic infection (15, 17, 20, 41). Here, we reveal a mechanism for these virulence defects: PrsA2 protects and folds the pore-forming toxin LLO, which is necessary for bacterial intracellular growth and pathogenesis (Fig. 3a through d). Secreted protein clients such as LLO need to be protected from aggregation at neutral pH to ensure maturation and function. Therefore, PrsA2 is likely interacting with unfolded protein clients in a highly dynamic manner. This hypothesis is supported by the binding of PrsA2 to the cholesterol-binding domain of LLO. Additionally, the Gram-positive *Staphylococcus aureus* PrsA and the distantly related Gram-negative *Escherichia coli* SurA have also been shown to possess anti-aggregation activity (42, 43), confirming a role of PrsA in protecting unfolded clients from aggregation while at the same time folding protein clients. Since several PrfA-dependent gene products including LLO are upregulated during infection, we speculate that PrsA2 sequesters and releases virulence factors that are required for phagosomal escape and replication within the host cytosol. However, biological cues, in addition to pH that could trigger their release from PrsA2, have yet to be determined. Because PrsA2 structure and function are well conserved in Gram-positive bacteria (44), we predict that other PrsA2 homologs similarly regulate the activity of pore-forming toxins and major virulence factors in Gram-positive bacteria.

Similar to the X-ray crystal structures of *Lm* PrsA1 (PrsA2 homolog) (30) and the Gram-positive *Bacillus subtilis* PrsA (45), the predicted PrsA2 structure also contains a PPIase domain (residues 135–228) and foldase domain composed of N (residues 20–134) and C (residues 229–293) terminal elements. PrsA2 activity depends on the foldase domain generated by dimerization (30) and the two PPIase domains (Fig. 3a through d; Table 2). The V91T mutation in the foldase pocket increased the affinity of PrsA2 for LLO, better protected LLO, and reduced LLO refolding kinetics (Table 3; Fig. 3a and b). We hypothesize that the foldase pocket is essential for binding hydrophobic unfolded or partially folded substrates, so mutations that impact the pocket dramatically affect chaperone activity. Furthermore, the affinity of PrsA2 V91T for LLO suggests the association rate between PrsA2 V91T and LLO is higher than the dissociation rate perhaps due to the formation of a stable protein complex at this pH level.

PrsA2 and homologous proteins dimerize via the N- and C-terminal domains of each protein monomer (30, 45). Consistent with this observation, PrsA2 is shown to form heat-resistant dimers in solution, suggesting PrsA2 dimers are essential under physiological conditions. PrsA2 dimerization is likely important for PrsA2 chaperone activity since the PrsA2 dimer forms a bowl-shaped pocket with dynamic PPIase domains that are shaped as rotational arms (Fig. 4c). Additionally, our previous findings suggest PrsA2 dimerization is important for ethanol resistance, integrity of the bacterial cell wall, and growth under osmotic and acidic conditions, consistent with a role in folding and secretion of protein factors (30). Like the Gram-negative SurA, which can function as a chaperone almost entirely without its PPIase domains (46, 47), PrsA2 PPIase activity is dispensable for LLO-dependent activity but only under certain conditions (e.g., pH 5, where LLO has optimal activity) (17). Our results demonstrate that PrsA2 PPIase activity is required for LLO-dependent activity at high pH and under conditions similar to the host cytosol (Table 2).

**Fig 5:**
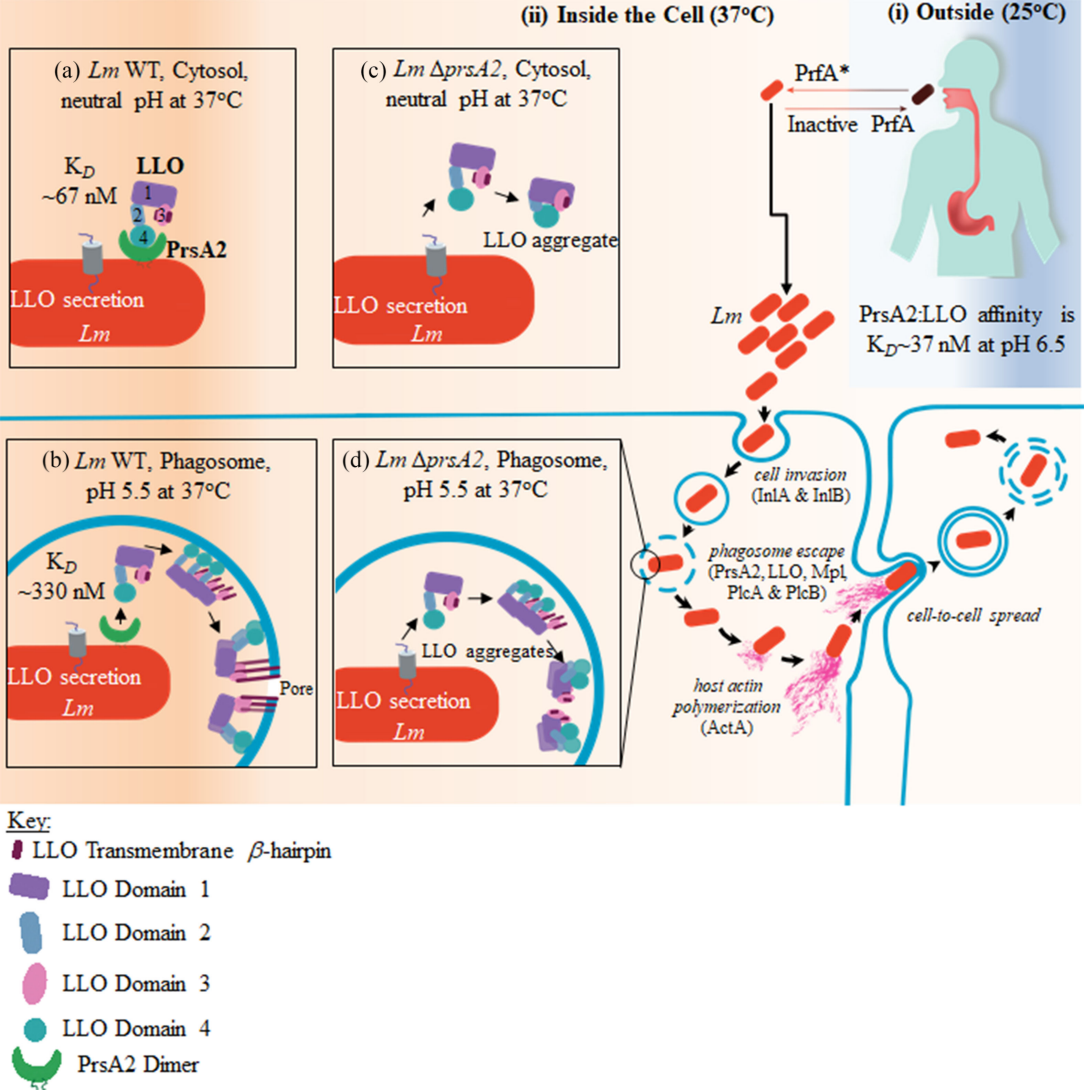
Model of *Lm* PrsA2 interacting with LLO in the environment and during active infection. (i) In a harsh environment (temperature ≤30°C) such as soil or vegetation, *Lm* exists as a saprophyte. When a susceptible individual ingests *Lm*, the bacteria become internalized. The switch from the saprophytic lifestyle to life inside the cell is controlled by the PrfA (reviewed in reference 9). In the environment, PrfA exists in a low-activity state. (ii) However, within the cell, PrfA becomes activated (PrfA*). PrsA2 and LLO are co-translated within the bacteria cell (20). (**a**) As *Lm* enters the cytosol (pH 7.4, 37°C), PrsA2 at the bacterial membrane folds, stabilizes, and sequesters functional LLO (high PrsA2-LLO affinity, *K*_*D*_ = ~67 nM), thereby preventing LLO aggregation. (**b**) The drop in pH (pH 5.5, 37°C) as *Lm* is internalized in a phagosome triggers the release of functional LLO by PrsA2 (low PrsA2-LLO affinity, *K*_*D*_ = ~330 nM) to bind and break down the phagosomal membrane, enabling *Lm* escape. (c and d) In the absence of PrsA2, secreted LLO at the bacterial membrane within the cytosol (pH 7.4, 37°C) forms irreversible aggregates due to the premature unfolding of the domain 3 transmembrane β-hairpins (12), leading to *Lm* that is deficient in escaping the phagosome. PrfA, positive regulatory factor A.

Based on the observed interactions between PrsA2 and LLO, we propose a model that considers the complete life cycle of *Lm* in the outside environment and during infection (Fig. 5a through d). The PrsA2 secretion profiles in the presence of prfA* suggest that several novel client proteins important for *Lm* physiology and virulence are regulated in a PrsA2-dependent manner (Fig. 1a through d); therefore, more studies are needed to define the PrsA2-client-protein repertoire. Furthermore, because protein-protein interactions occur as highly dynamic processes on rapid timescales, we expect atomic-level techniques such as nuclear magnetic resonance will be required to better understand how PrsA2 binds, holds, and folds LLO.

In summary, we have determined that the PrsA2 chaperone is critical for regulating the pore-forming toxin LLO under infection-relevant conditions. We show that PrsA2 physically interacts with and accelerates LLO folding. Lastly, we predict that PrsA2 protects and stabilizes LLO via binding across stable hydrogen bonds, salt bridges, and a large hydrophobic interaction surface. These findings present a new model of how membrane-anchored bacterial chaperones stabilize, fold, and regulate the activity of key virulence factors in Gram-positive bacteria.

## MATERIALS AND METHODS

### Bacterial strains, plasmids, and media

Bacterial strains and plasmids used in this study are listed in Table S4. Genetic manipulations were done in the background of the *Lm* 10403S wild-type strain (NF-L100). The constitutive Lm prfA* mutant contains an in-frame *prfA* deletion and is complemented with a chromosomal integrated plasmid, pPL2-*prfA* (L140F), that confers the prfA* phenotype (NF-L1167) (13). The Lm prfA*Δ*prsA2* strain was generated by replacing the *prsA2* open reading frame with an erythromycin resistance cassette (erm) in the NF-L1167 background (NF-L1637) (16). The generation of *Lm* Δ*prsA2* (NF-L1651)(16) and other *Lm prsA2* mutants [Δ*prsA2 + pPL2-prsA2* (NF-L1656), Δ*prsA2 + pPL2-prsA2* (V91T) (NF-L3804), Δ*prsA2 + pPL2-prsA2* (N+C) (NF-L1674), Δ*prsA2 + pPL2-prsA2* (no dimer/*prsA2^M^*) (NF-L3806)] has also been described previously (16, 17, 30). Unless otherwise noted, *Lm* and *Escherichia coli* strains were propagated in brain heart infusion (BHI) broth (Difco) and Luria broth (LB) (Invitrogen), respectively, at 37°C.

### Cell fractionation

This method was adapted from reference 48 with some modifications (48). Briefly, to assess the contribution of PrsA2 to protein secretion during the exponential phase when PrfA is constitutively active, bacterial cultures were grown to mid-log phase optical density at 600 nm (OD_600_) of 0.6, normalized, and then fractionated for cell wall and released proteins. Bacterial cells were pelleted at 8,000 rpm for 15 min at 12°C. To collect released proteins, supernatants were recovered, and trichloroacetic acid (TCA) precipitated at a final concentration of 10% (vol/vol). TCA-treated supernatants were incubated on ice for 60 min, and precipitated proteins were pelleted by centrifugation at 8,000 rpm for 15 min at 12°C. Supernatants were discarded; recovered proteins were washed with ice-cold acetone; and protein pellets were collected by centrifugation, air-dried, and resuspended in buffer M (0.5-M sucrose, 50 mM Tris-HCl, pH 8, 10 mM MgCl_2_, 2 µL protease inhibitor cocktail 3 (Millipore-Sigma), and 3 mM Na azide]. To collect the cell wall fraction, cell pellets were washed with buffer (50 mM Tris-HCl, pH 8, and 10 mM MgCl_2_) and resuspended in isotonic buffer M to prevent protoplast lysis and contained 3 mM Na azide, which blocks protein secretion (48). Then the bacterial cell wall was digested with the addition of 20-µg/mL *Listeria*-specific phage endolysin A118, 100-mg/mL lysozyme and incubation at 37°C for 15 min. The formation of cell protoplasts was monitored by microscopy. Next, the cell wall fraction was separated from the protoplasts by centrifugation at 15,000 × *g* for 3 min at 12°C, where the resulting supernatant contained the cell wall fraction. Cell fractions were probed with antibodies specific for PrsA2 (16) and InlA (a kind gift from Dr. Keith Ireton at the University of Otago) (Fig. S8a and b). PrsA2 is cell associated and also released (16), whereas InlA is covalently linked to the cell wall (48–50).

### TMT-MS

TMT-MS was done at the University of Illinois at Chicago Mass Spectrometry, Metabolomics, and Proteomics Facility (MMPF). At the MMPF, protein samples were taken through filter-aided sample preparation. Briefly, 50 µg of protein samples was dissolved using 8 M urea in 0.1 M Tris-HCl, pH 8.5, and then filtered with a 0.22-µm membrane (Millipore). Flow-through was collected and transferred into a 1.5-mL MicroconYM-10 centrifugal unit (Millipore). Protein reduction, alkylation, and tryptic digestion were carried out step by step by using a centrifugal unit. The peptide samples were digested overnight at 37°C and then eluted twice with 50 µL 0.1% formic acid (FA). Peptide concentration at every step was determined using a Nanodrop ONE (Thermo Scientific). Digested peptides were desalted, dried, and stored at −80°C until further use. For sample labeling, 100 µg of peptides per sample for 10-plex TMT was isobaric-labeled according to the manufacturer’s instructions (Thermo Scientific). After sample labeling, excess reagents and detergents were removed by reversed-phase solid-phase extraction (Oasis HLB C18 SPE, Waters Corp.). Next, peptide samples were dried, and resulting pellets were stored at −80°C for further processing. Samples were fractionated by high-pH reversed-phase chromatography using Agilent 1260 HPLC and Waters xbridge column (c18 4.6 × 150 mm, 3.5 µm). Ninety fractions were collected and combined into 10 fractions then desalted using Nestgroup c18 tips (Southborough). The fractionated peptides were further dried and redissolved in 0.1% FA for liquid chromatography (LC)-tandem mass spectrometry (MS/MS) analysis. Peptide fractions were run on the Thermo Fisher Orbitrap Velos Pro coupled with Agilent NanoLC system over a 1-hour gradient using LC columns (15 cm × 75 µm ID, Zorbax 300SB-C18) (Agilent). Peptide samples were analyzed with a 1-hour linear gradient (0%–35% acetonitrile with 0.1% formic acid), and data were obtained in a data-dependent manner, in which MS/MS fragmentation is performed on the top 12 intense peaks of every complete MS scan. The generated RAW files were converted into .mgf files using MSConvert (from ProteoWizard). Database protein search was performed using the Mascot server (Matrix Science). For quantitative analysis, search results from 18 runs were imported into Scaffold (Proteome Software).

### Tandem mass tagged-mass spectrometry data analysis

TMT-MS data analysis was performed using readings from two independent replicates of *Lm* cell wall (secreted) and released (supernatant) fractions with *Lm* strains prfA* (NF-L1167) and prfA*Δ*prsA2* (NF-L1637). To account for variations within samples, we used Pearson’s correlation analysis, which demonstrates a positive correlation between replicates (Fig. S9a through d). Raw data, in log_2_ form were linearized and normalized to the protein concentration of the control conditions; Lm prfA*Δ*prsA2* was normalized to the Lm prfA* strain. The fold change of proteins in each experimental condition was determined by dividing the normalized values in the experimental condition by the corresponding normalized values in the control condition. Data were filtered for duplicated gene names, proteins with missing values and “contaminant proteins” (which we defined as proteins derived from the BHI media used to grown the strains and decoy proteins). Contaminant protein hits in the data were indicated by the symbol “+” and were filtered using the filter function. Proteins detected in at least one out of two replicates were identified, and replicates in the same condition were screened for similar secretion levels. Further analysis using differential enrichment based on linear models and empirical Bayes statistics and rendering were done in R software (v.4.3.0) (51) using the DEP package (25). The full table of TMT-MS data (Table S5) and the data analysis scripts are provided (https://github.com/Selalichs/LmPrsA2).

### Co-immunoprecipitation

From saturated overnight cultures of Lm prfA* (NF-L1167), prfA*Δ*prsA2* (NF-L1637), and prfA*Δ*prsA2 + pPL2-prsA2* (NF-L1659), and *Lm* wild type (NF-L100)*,* Δ*prsA2* (NF-L1651), and Δ*prsA2 + pPL2-prsA2* (NF-L1656) grown in LB broth, a 1:20 dilution in fresh LB broth of each strain was grown ~5 hours, conditions where *Lm* secretes proteins including PrsA2 and LLO (15, 41, 44). The bacterial OD_600_ was determined, and cells were normalized to an OD_600_ of 0.8/mL. Protease inhibitor cocktail 3 (Millipore-Sigma) was added to 10 mL of normalized bacterial culture followed by centrifugation at 13,000 rpm for 5 min. The supernatant (containing secreted/released proteins including PrsA2 and LLO) was passed through a 0.22-µm filter and concentrated to 1 mL using the Centricon 3 (Sigma-Aldrich). Then, 2.5 µL of anti-LLO antibody was added and incubated at 4°C overnight. Sepharose beads (Sigma-Aldrich) (10 mg) were added in radioimmunoprecipitation assay (RIPA) buffer (150 mM NaCl, 50 mM Tris-HCl, pH 8.0, 10 mM EDTA, 1% NP-40, 0.5% deoxycholate, 0.1% SDS, and 250 µM phenylmethylsulfonyl fluoride (PMSF) to samples and incubated for 2.5 hours. The slurries were loaded into immunoprecipitation columns (Pierce) and were washed twice with 500 µL RIPA buffer. Beads were recovered from the columns and resuspended in 80 µL of 2× SDS boiling buffer and boiled for 6 min. Then, 20 µL of each sample was resolved on a 12% gradient SDS polyacrylamide gel and probed with anti-LLO (1:2,500 dilution) and anti-PrsA2 (1:2,500 dilution) antibodies (16). Secondary antibody conjugated to horseradish peroxidase (HRP) (1:2,500 dilution) was detected by ECL kit (Thermo Scientific). Data show a representation of three independent replicates.

### Gene cloning, site-directed mutagenesis, protein expression, and purification

The expression constructs for PrsA2 (pQE60-*prsA2*) and the PrsA2 N+C mutant [pQE60-*prsA2* (N+C)] were generated previously by reference (16) without the signal sequence (starting at amino acid 21) (Table S4) (16). The *hly* gene that encodes LLO from strain *Lm* 10403S was PCR amplified without the signal sequence (25–529 amino acids) from genomic DNA using primers in Table S6 and was cloned into the pQE30 expression vector containing an N-terminal 6× His tag. For expression constructs of site-directed mutants—PrsA2 V91T and PrsA2 No dimer mutant*/*PrsA2^M^ (V28A/L40A/Y41A/M44A), point mutations were introduced into the pQE60-*prsA2* plasmid using primers in Table S6. Briefly, using pQE60-*prsA2* plasmid as a template, site-directed mutations were introduced using the QuickChange Lightning site-directed mutagenesis kit (Agilent Technologies). Once mutations were confirmed by sequencing analysis, recombinant protein expression was performed by transforming the plasmids into *E. coli* BL21 (DE3*) cells (52). Starting overnight cultures were grown in LB supplemented with 50 µg/mL of carbenicillin with vigorous shaking at 37°C and 200 rpm. Next, overnight cultures were used for inoculating 1 L of LB supplemented with 50 µg/mL of carbenicillin at 1:200 dilution, at 37°C, and at 200 rpm. Expression of recombinant PrsA2, PrsA2 mutants, and LLO was induced at an OD_600_ of 0.6 by adding 0.8 mM (for PrsA2 strains) and 0.1 mM (for the LLO strain) of isopropyl β-d-1-thiogalactopyranoside (17), and grown for 12 hours at 30°C and 200 rpm. Cells were pelleted by centrifugation at 8,000 rpm, at 4°C for 45 min, flash frozen, and stored at −80°C for at least 24 hours. For purification, frozen pellets were thawed on ice and resuspended in chilled lysis buffer (50 mM Tris, pH 7.5, 500 mM NaCl, and 25 mM imidazole) containing protease inhibitor cocktail 3 (Millipore-Sigma) and DNase I (Millipore-Sigma). Resuspended cells were sonicated at 70% amplitude using 10 s by 10 cycles for 5 min at 4°C. The lysates were clarified by centrifugation at 8,000 rpm for 40 min at 4°C, and the supernatants were collected. The supernatants were passed through a nickel-NTA column washed with two column volumes of lysis buffer, after which the column was washed again with 10 column volumes of lysis buffer. Recombinant proteins were eluted with elution buffer (50 mM Tris, pH 7.5, 500 mM NaCl, and 500 mM imidazole) and dialyzed overnight at 4°C in a dialysis buffer [20 mM 2-(N-morpholino)ethanesulfonic acid (MES), pH 5.5, 6.5, or 7.4; 100 mM NaCl, 1 mM β-mercaptoethanol, and 10% glycerol). Dialyzed proteins were analyzed by SDS-PAGE and stained with Coomassie dye for purity (Fig. S10), and protein concentrations were determined by the bicinchoninic acid assay (Pierce).

### Circular dichroism spectroscopy

Circular dichroism spectra data were collected on the OLIS 17 UV/VIS/NIR spectrometer at 25°C using a 0.2-mm path length cuvette from 190- to 260-nm wavelength. Protein samples were diluted in 20 mM KH_2_PO_4_, pH 7.4, at 0.05-mg/mL concentrations. Data were normalized to the buffer, and the average of three scans was collected using OLIS software. Data were visualized using the OLIS analysis software package and plotted with the GraphPad Prism (v.9.5.1).

### Protein dimer formation assay

Purified recombinant His-tagged PrsA2 and PrsA2 No dimer mutant/PrsA2^M^ (500 ng) were incubated with 10 µM final concentration of BS3 bis-(sulfosuccinimidyl)-substrate crosslinker for 1 hour at room temperature in buffer Q (20 mM HEPES, 200 mM NaCl, pH 6.5). Reactions were heated at 85°C for 10 min in 2× SDS boiling buffer with β-mercaptoethanol followed by separation on a 12% gradient gel by SDS-PAGE for 1 hour. Gels were transferred to polyvinylidene fluoride (PVDF) membrane and probed with an anti-His primary antibody (1:1,000) and secondary antibody conjugated to alkaline phosphatase (1:5,000) (Thermo Fisher). Protein bands were visualized using a one-step NBT/BCIP substrate (Thermo Fisher).

### Protein labeling, MST, and data analysis

Recombinant proteins were labeled using the NanoTemper 2nd Generation Red NHS Dye (Nanotemper Technologies) that binds to the primary amines of protein chains to form stable dye-protein conjugates. Protein samples (5–20 µM) were passed through buffer exchange columns to remove incompatible buffer components. Solid NHS dye was dissolved in 100% dimethyl sulfoxide (DMSO) and diluted to 300 µM using labeling buffer (pH 8.2–8.5). Next, the protein sample was mixed with a two- to threefold excess of reconstituted dye and incubated in the dark for 30 min at room temperature. Excess unreacted NHS dye was removed by passing the reaction through a gel filtration column (Nanotemper Technologies). The degree of label (DOL) of the labeled protein was determined using the formula *A*_650_/195,000/M/cm × concentration of labeled protein, where *A*_650_ = absorbance at 650 nm, and 195,000/M/cm is the molar absorbance of the red NHS dye, and DOL of 0.5–1.0 was considered optimal. Labeled protein samples were aliquoted in small volumes, flash frozen, and stored at −80°C until further use.

For the MST experiment, labeled protein, unlabeled protein, and buffer (20 mM MES, 100-mM NaCl, 1 mM β-mercaptoethanol, 10% glycerol, supplemented with 0.05% Tween 20) were kept at the same pH (pH values of 5.5, 6.5, or 7.4). To test binding, we performed serial dilution of the unlabeled protein (4–35 µM) in 10-µL volume in low-binding tubes. Equal concentrations (20–150 nM) of 10 µL labeled protein were added to the serially diluted unlabeled proteins and incubated at room temperature for 5 min. Samples were then loaded in capillary tubes (Nanotemper Technologies), and thermophoresis was monitored using the Microscale Thermophoresis machine (NanoTemper Monolith NT.115) at 20%–60% excitation power and 50% MST Power at 25°C or 37°C with MO.control v.1.6.1.

All data were analyzed using the PALMIST (27) and GUSSI (28) pipeline. Briefly, MST data sets from the MO.control software were imported into the PALMIST software package. Fluorescence time traces were normalized based on the mean fluorescence values. A 1:1 *K*_*D*_ fitting parameter was applied with a 95% confidence interval, and the *K*_*D*_ values are presented in the form of “*K*_*D*_ (lower limit, upper limit).” Data were examined for kinetic effects to ensure that thermophoresis had achieved equilibrium. Unbound thermophoresis is subtracted from the data sets. A preset T-jump is used to calculate thermophoresis, and resulting *K*_*D*_ values are compared to *K*_*D*_ values from the MST MO.affinity analysis software. Analyzed results were exported and rendered using GUSSI. The illustrated figures from GUSSI are presented in the form of three panels. The top, middle, and lower panels represent the fluorescence time traces, binding curve, and residuals (a plot between the fitted line and the data), respectively. Error bars represent the standard deviations of at least three independent replicates.

### ITC

The thermodynamic interaction between the recombinant proteins was measured using the Affinity ITC LV machine (TA instruments) at 25°C or 37°C. Recombinant protein samples were thoroughly dialyzed with the ITC buffer (20 mM MES, 100 mM NaCl, 1 mM β-mercaptoethanol, 10% glycerol, and pH values of 5.5, 6.5, or 7.4) at 4°C overnight. Frozen protein samples were thawed to room temperature and degassed under vacuum for 15 min. An approximately 20- to 50-fold higher concentration of the titrants was injected into the titrands for 20 injections with an injection volume of 2–5 µL for 300–400 s, stirring rate of 125 rpm, and reference power 5 µcal/s using ITCRun (v.3.8.0) (TA Instruments). Raw heat rates were corrected by subtracting the heat of dilution from the control experiment or average of the final few injections, with the first injections excluded. The binding thermograms for all samples were fitted with the independent binding model using NanoAnalyze software (v.2.2.0, TA Instruments).

### Dynamic light scattering

Dynamic light scattering was performed using the DynaPro NanoStar (Wyatt Technology). Recombinant LLO protein samples (5 µm) at pH values of 5.5, 6.5, and 7.4 were filtered using a 0.22-µm filter membrane (Foxx Life Sciences). Bovine serum albumin (~30 µM) supplemented with 0.9% NaCl solution containing sodium azide was used as a control (Thermo Scientific). Measurements were made at 37°C and carried out in a quartz cuvette (Wyatt Technology) in volumes of 45 µL over 15 min. The acquisition time (10 s) and number (10–20) were determined using the optimization calculator on the instrument software Dynamics (v.7.5.0.17), with lysozyme data as reference. Data were collected using the following parameters: MW-R model, globular proteins; dn/dc (mL/g), 0.185; Rg model, sphere; A2 (mol mL/g^2^), 0.0; internal Standard Rh (nm), 50.0. Data were processed by the Dynamics Software, and at least three replicates were performed for each sample.

### PrsA2-protection assay

Purified LLO protein was diluted into ice-cold buffer C (125 mM NaCl, 1.706 mM Na_2_HPO_4_·7H_2_O, 33.29 mM H_2_NaO_4_P·H_2_O, 1 mM dithiothreitol [DTT]) at pH 7.4 to a final concentration of 100 nM. Ice-cold, purified, concentrated recombinant PrsA2 protein was added into the LLO solution at a 30-fold molar excess (3 µM), and both proteins were mixed uniformly and incubated on ice at pH 7.4 for 15 min to allow for the interaction between PrsA2 and LLO to occur. As a negative and molecular crowding control, PrsA2 was omitted from the LLO sample, and its volume was substituted with buffer C, pH 7.4. To confirm that PrsA2 alone does not have the ability to lyse the Alsevers sheep’s RBCs, a PrsA2-only control was used. The LLO/PrsA2 protein mix was subsequently transferred to a 37°C water bath and incubated for 1 hour to induce LLO aggregation and precipitation at unfavorable pH and temperature, in the presence or absence of PrsA2. The mix was then cooled on ice and serially diluted in ice-cold buffer C, pH 5.5. Next, 50-µL aliquots of serially diluted LLO/PrsA2 protein solution or control solutions were added to each well of a 96-well plate, followed by the addition of 150 µL of 3× washed 1.33% sheep’s RBCs (Cocalico Biologicals) in Alsevers solution at pH 5.5, supplemented with 1-mM DTT, for a total volume of 200 µL per well. The plate was incubated at 30°C for 30 min to allow for LLO-mediated hemolysis of RBCs to occur. Hemolysis was stopped by cooling the 96-well plate on ice for 5 min and then centrifuging it in a swing bucket rotor (IEC CL31R Multispeed Centrifuge, Thermo Scientific) at 1,800 rpm for 5 min at 4°C, to spin down any remaining whole RBCs and cellular debris. One hundred fifty microliters of the resulting supernatant was taken from each sample and transferred to a new 96-well plate, and the extent of hemolysis was assessed by measuring absorbance at 415 nm of hemoglobin released from RBCs as the result of LLO-mediated hemolysis. For 100% hemolysis control, RBCs were lysed by passing cells five times through a 22-gauge needle and then spun down to remove cellular debris. The hemoglobin absorbance at 415 nm was converted to corresponding LLO activity using hemolytic units, with one unit defined as the reciprocal of the lowest LLO concentration that results in 50% hemolysis of RBCs.

### Chaperone-assisted protein folding assay

Purified recombinant LLO was denatured by incubating in a denaturing buffer [8 M urea, 30 mM 3-(N-morpholino)propanesulfonic acid (MOPS)-HCl, 1 mM β-mercaptoethanol, pH 6.5] (53) for 45 min at room temperature. Denatured LLO was then diluted 1:20 in 1× phosphate buffered saline (PBS)-1 mM DTT buffer (0.137 M NaCl, 0.0027 M KCl, 0.01 M Na_2_HPO_4_, KH_2_PO_4_, 1 mM DTT, pH 6.5). Diluted LLO (~0.01 µM) was incubated in the presence and absence of recombinant PrsA2 or PrsA2 mutants in a final volume of 100 µL for 5 min. Denatured LLO alone control (α, 0.01 µM) was prepared by diluting urea-denatured LLO 1:20 with 1× PBS-1 mM DTT buffer, while the wild-type non-denatured LLO control (Ψ, 0.01 µM) was diluted 1:20 with 1× PBS-1 mM DTT buffer. Next, the hemolytic activity of refolded LLO was measured using Alsevers sheep’s RBCs (54). One hundred microliters of 5% sheep’s RBCs was added and incubated for 45 min at room temperature. The amount of released hemoglobin by refolded and functional LLO was determined after centrifugation and collection of supernatants for absorbance measurement in a flat-bottomed 96-well plate. Released hemoglobin was determined by measuring the absorbance at 450 nm using the Biotek Cytation five-plate reader (Agilent Technologies). The amount of LLO required to lyse 50% of the sheep’s RBCs was determined and compared to non-denatured LLO. One hundred percent lysis of the sheep’s RBCs was determined by incubating 0.8-µM non-denatured LLO and 1% Triton-X-100 with the RBCs at room temerature. The hemolytic activity was determined by measuring the amount of toxin required to lyse 50% of red blood cells and dividing the absorbance by the non-denatured LLO at the same concentration used in the assay. The LLO refolding experiment over time was performed similarly to the above with minor modifications. Briefly, urea-denatured LLO was diluted 20-fold, and PrsA2 and PrsA2 mutants were added and incubated for 5 min. The LLO-dependent hemolytic activity was measured from the time of the addition of Alsevers sheep’s RBCs to 140 min. Assays were performed in triplicate for PrsA2 and PrsA2 mutants, and the standard error of the mean was determined.

### Hemolytic activity of *Lm* strains

Hemolytic activity was measured as previously described with few modifications (17). Briefly, *Lm* cultures (3 mL) were grown overnight in LB. Then, these cultures were diluted (1:10) in fresh LB broth and grown for ~5.5 hours with shaking at 37°C and 180 rpm because secreted LLO is detectable in the culture medium at this time point (15, 44). Bacterial cells were normalized based on the optical density at 600 nm, and supernatants were collected after centrifugation. Sample supernatants were then serially diluted with 1× PBS containing 1 mM DTT at pH values of 5.5, 6.5, or 7.4 and incubated at 37°C for 30 min. One hundred microliters of washed 20% Alsevers sheep’s RBCs was added to the supernatants, followed by a second incubation at 37°C for 30 min. The resulting mixtures were pelleted by centrifugation at maximum speed, and the dilution resulting in 50% RBC lysis was determined by visual inspection. Hemolytic units were determined as the reciprocal of the dilution resulting in 50% RBC lysis. The fold reduction was determined by dividing the hemolytic unit of each mutant by wild-type value, and the resulting fraction was subtracted from one. The hemolytic assay was performed twice using three independent replicates.

### Structure prediction and modeling

AlphaFold-multimer (55) was used to predict the structure of the PrsA2-LLO multimer from PrsA2 and LLO sequences lacking their respective signal sequences (PrsA2, amino acids 21–293; LLO, amino acids 25–529). The predicted structures were processed using the Schrödinger Maestro Release 2023-2 protein preparation wizard (default parameters) to assign protonation states, slightly minimize the structure geometry, etc. Bonds between the predicted structures were identified using LigPlot+ (56) with the following parameters: max hydrogen-acceptor distance 2.70 Å and maximum donor-acceptor distance 3.35 Å. Protein-structure images were rendered using MacPyMOL (v.2.5.4).

### Statistical analysis

Unless otherwise noted, data were analyzed using Prism software (v.9; GraphPad Software, Inc.). PyMol (v.2.5.4; Schrödinger, LLC) was used to generate all protein structures, and conclusions were based on a type I error rate of 0.05 (57).

## SUPPLEMENTAL MATERIAL

Supplemental Figures mbio.00743-24-s0001.pdf Figures S1-S10.

Supplemental material mbio.00743-24-s0002.pdf Supplemental figure legends and tables.

ASM does not own the copyrights to Supplemental Material that may be linked to, or accessed through, an article. The authors have granted ASM a non-exclusive, world-wide license to publish the Supplemental Material files. Please contact the corresponding author directly for reuse.
