# Supplementary figures and images for: The chaperone PrsA2 regulates the secretion, stability, and folding of listeriolysin O during Listeria monocytogenes infection
Source: mBio. 2024 May 29;15(7):e00743-24. doi: 10.1128/mbio.00743-24 (PMC11253611; doi:10.1128/mbio.00743-24)

Fig. S1

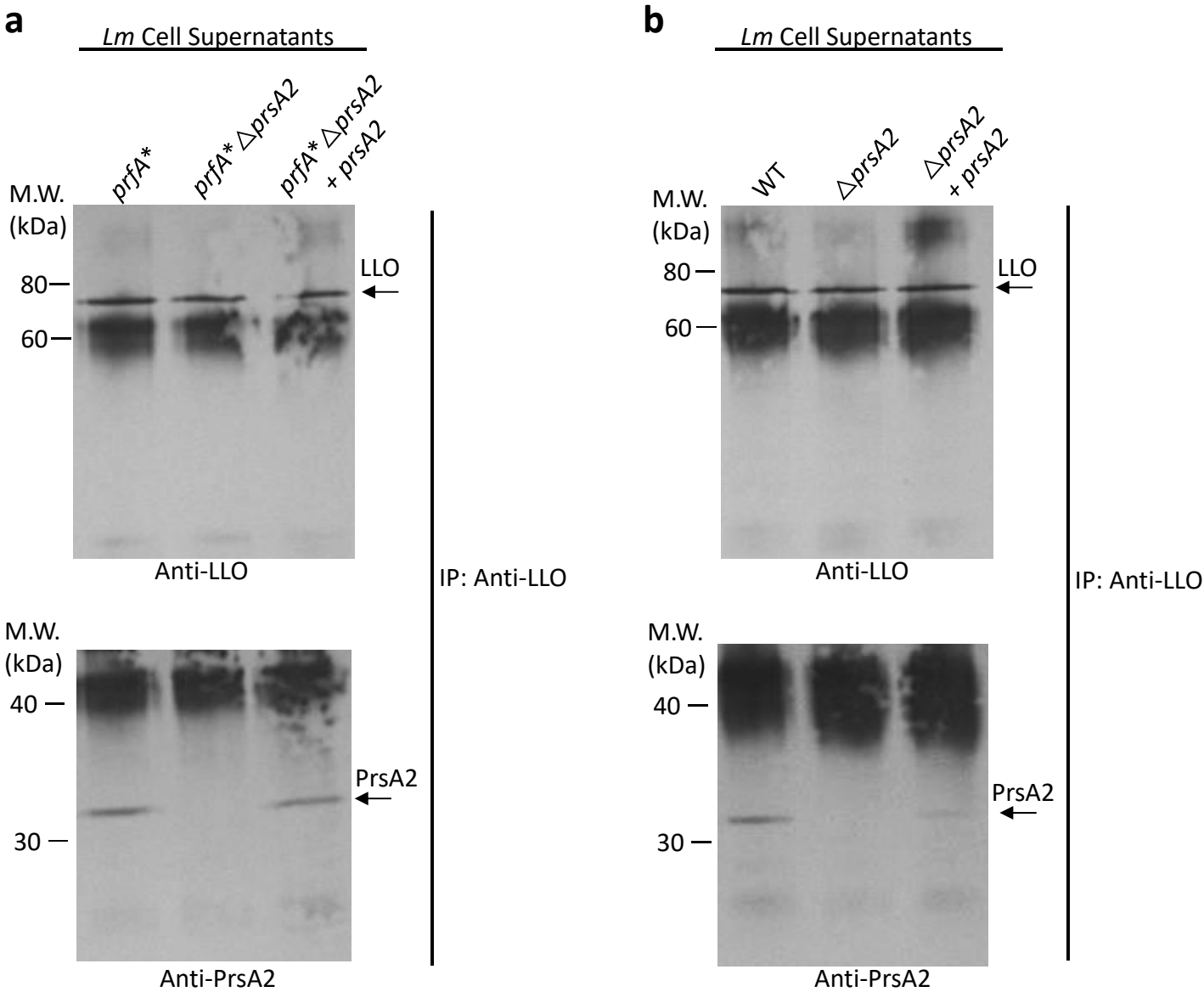

**Fig. S2**

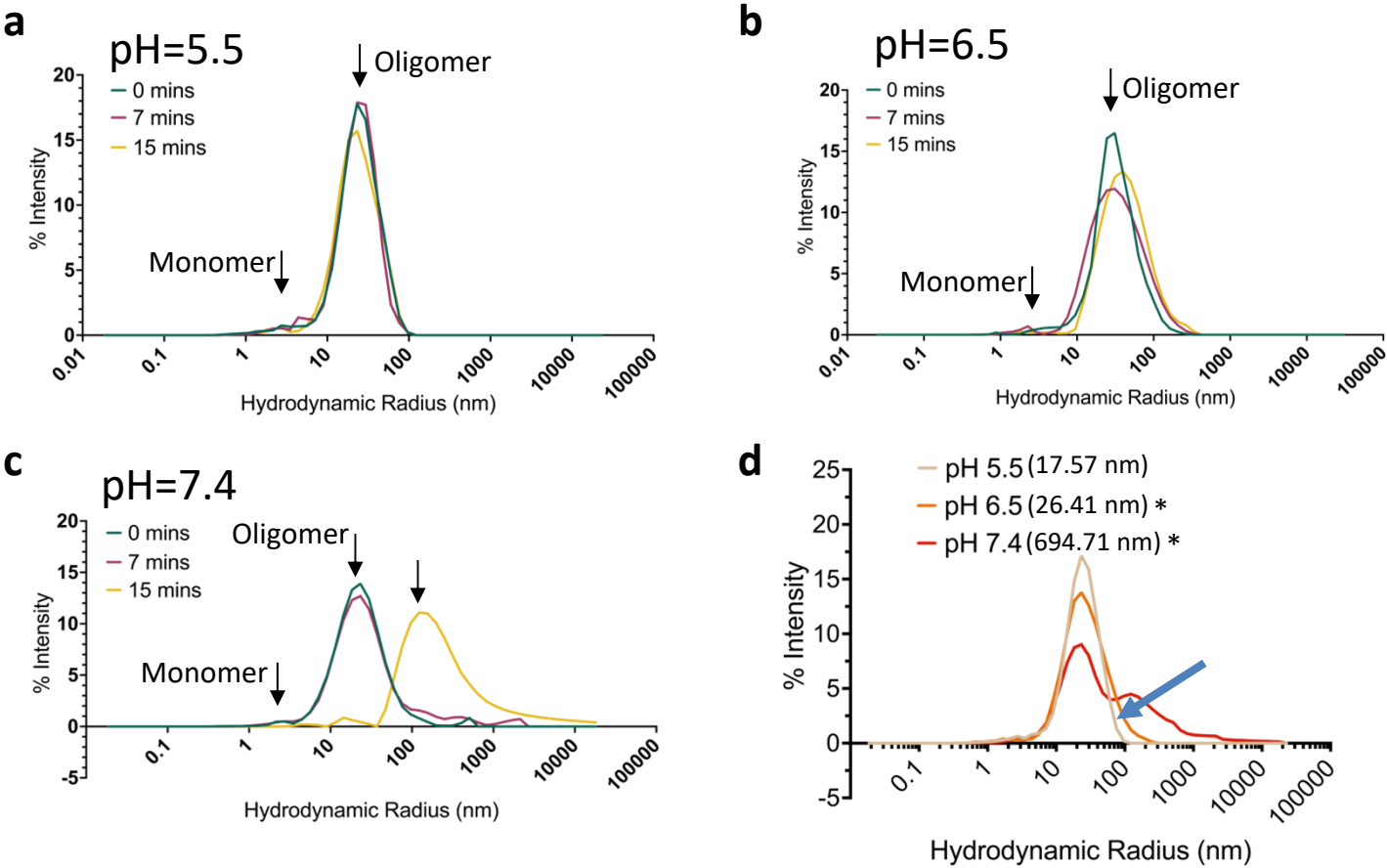

**Fig. S3**

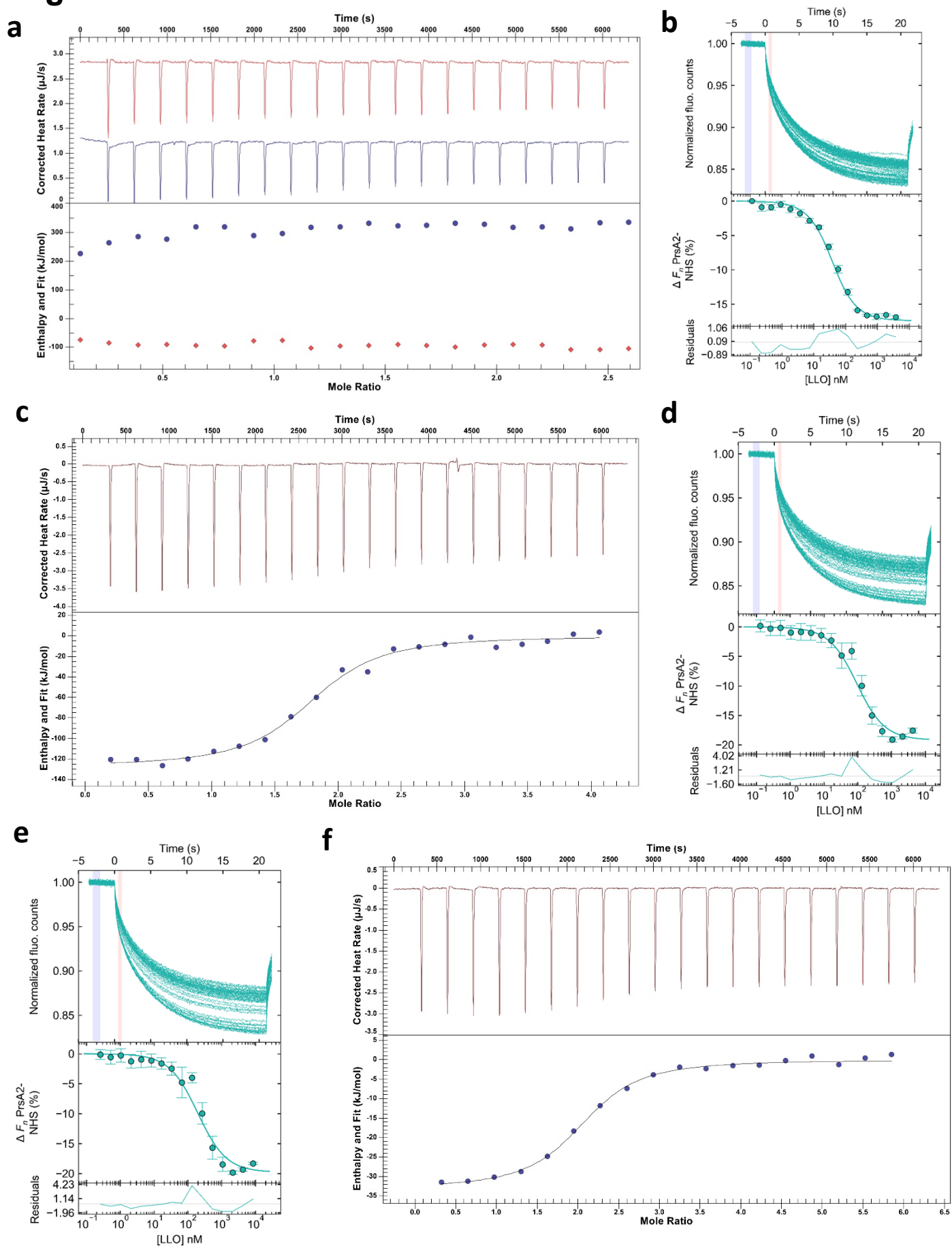

**Fig. S3**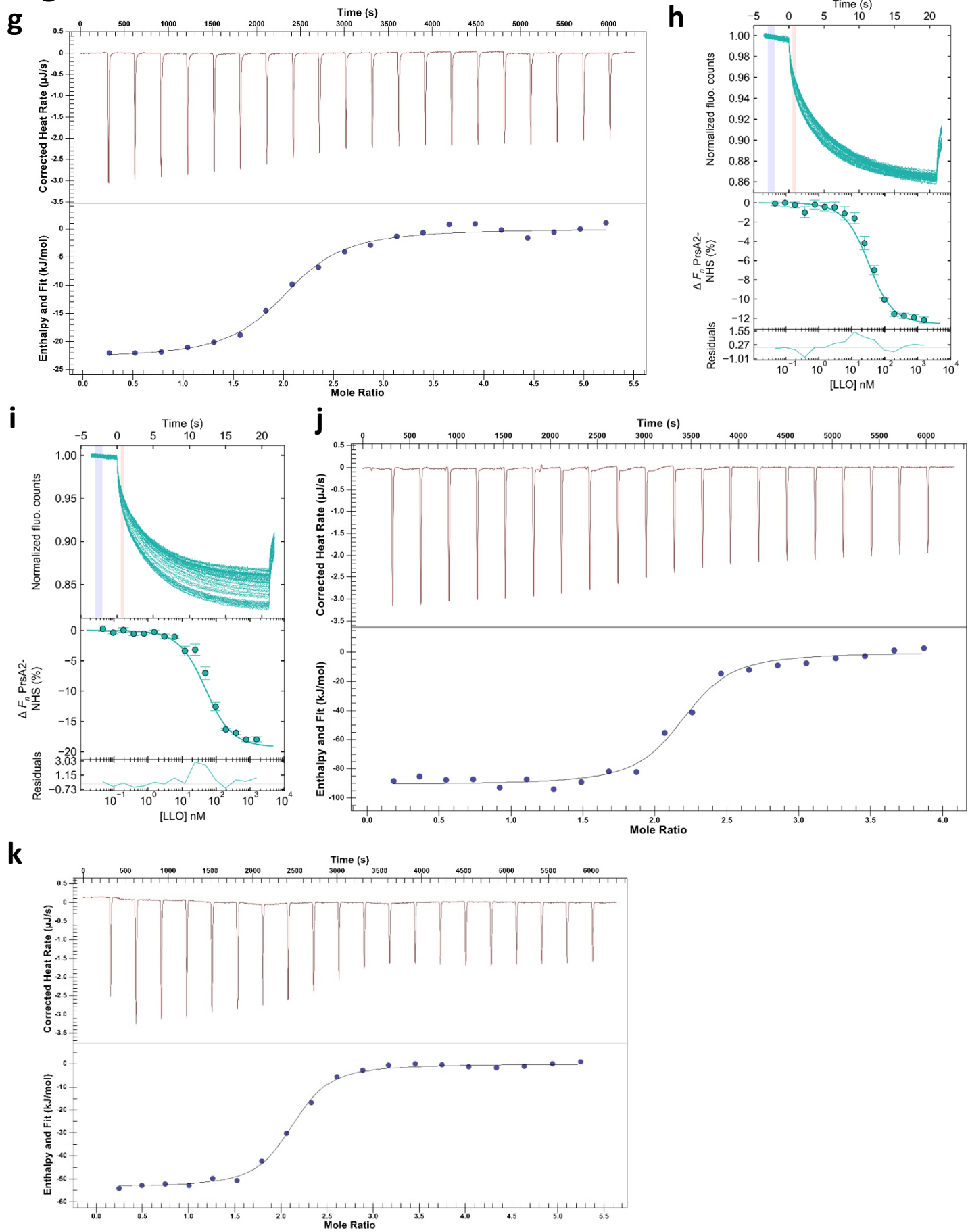

**Fig. S4**

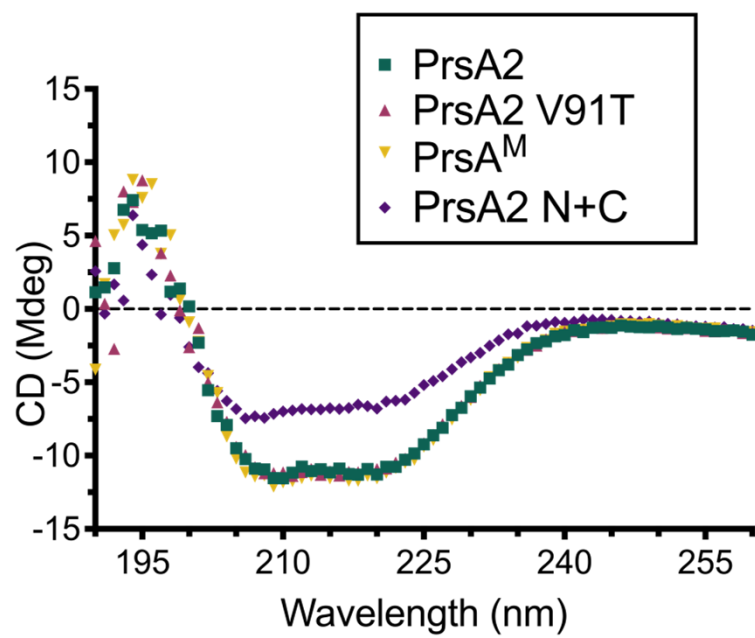

Fig. S5

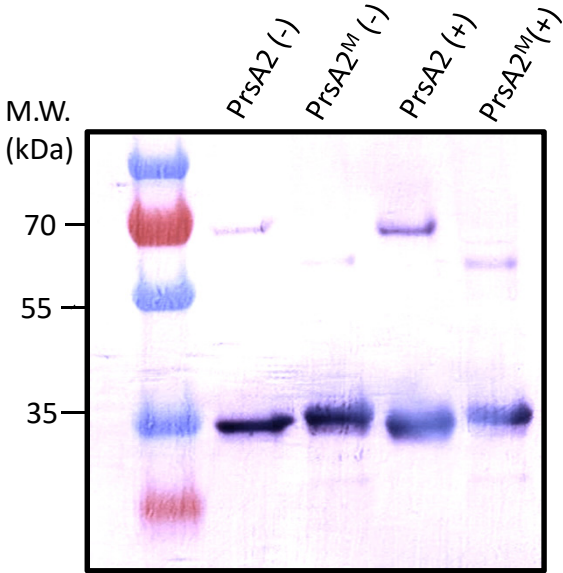

**Fig. S6**

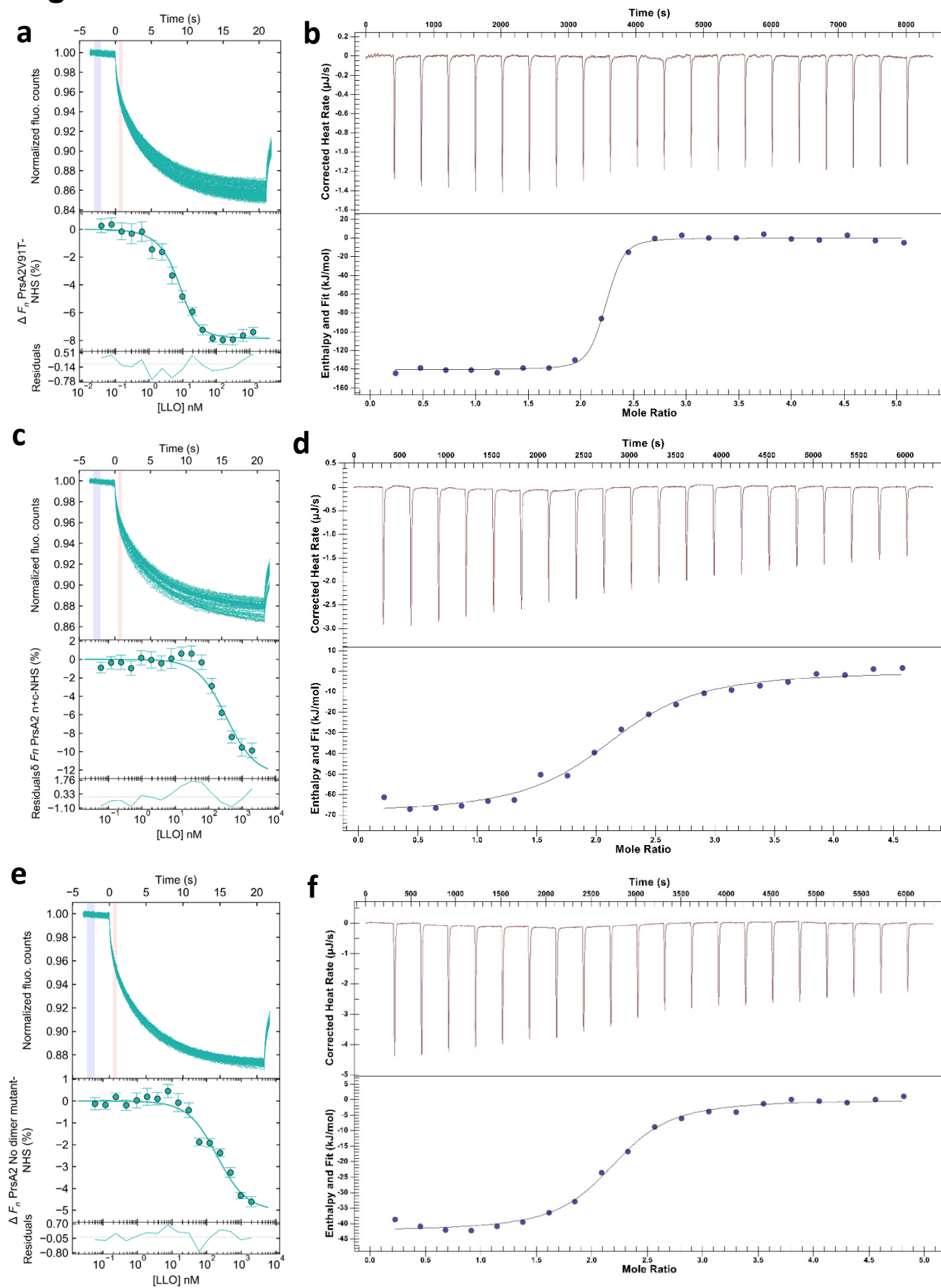

Fig. S7

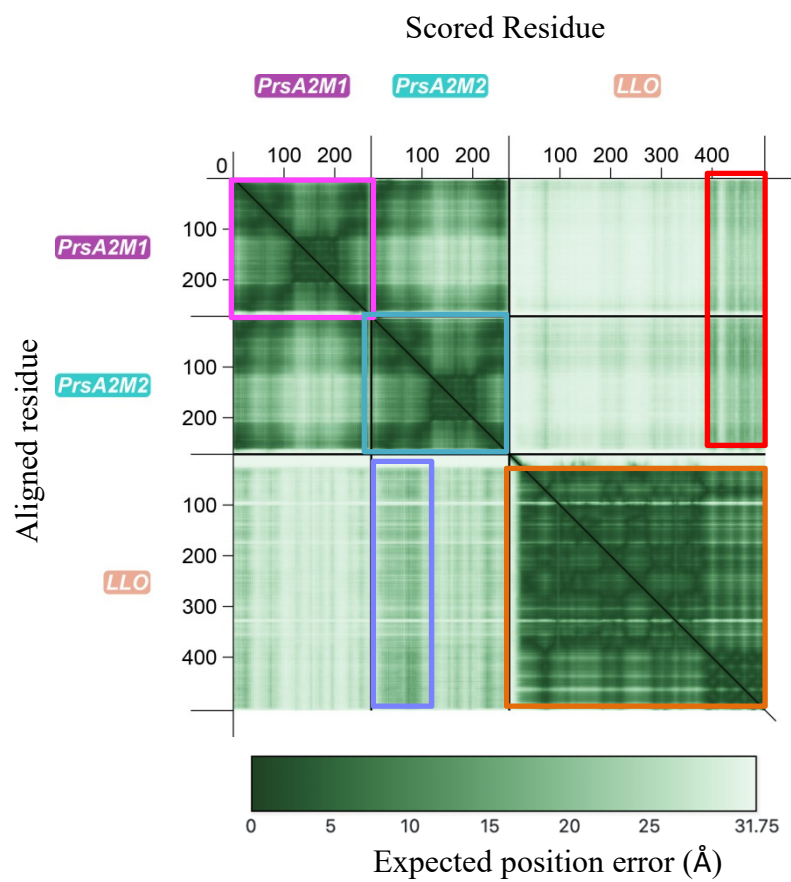

**Fig. S8**

**a**

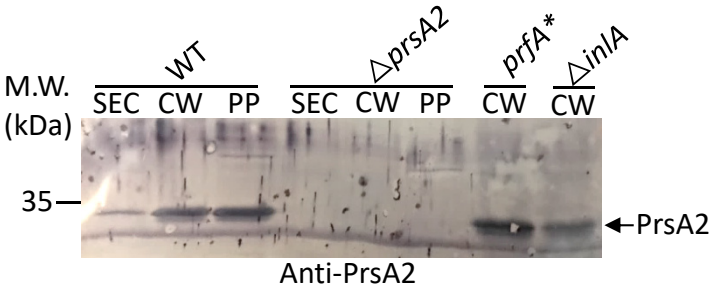

**b**

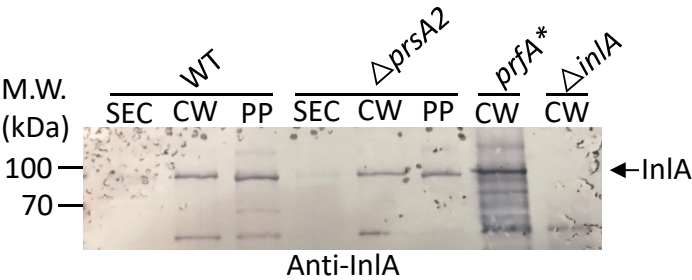

Fig. S9

a

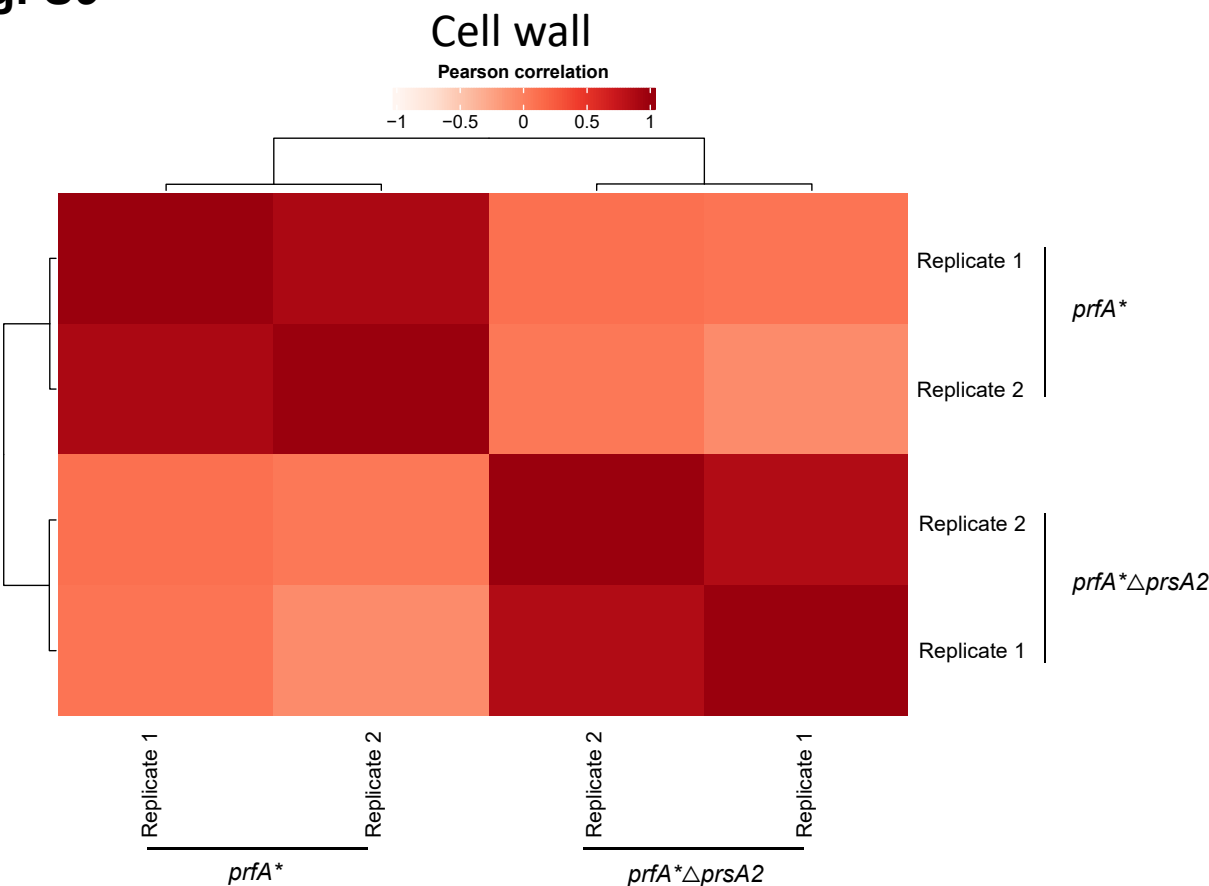

b

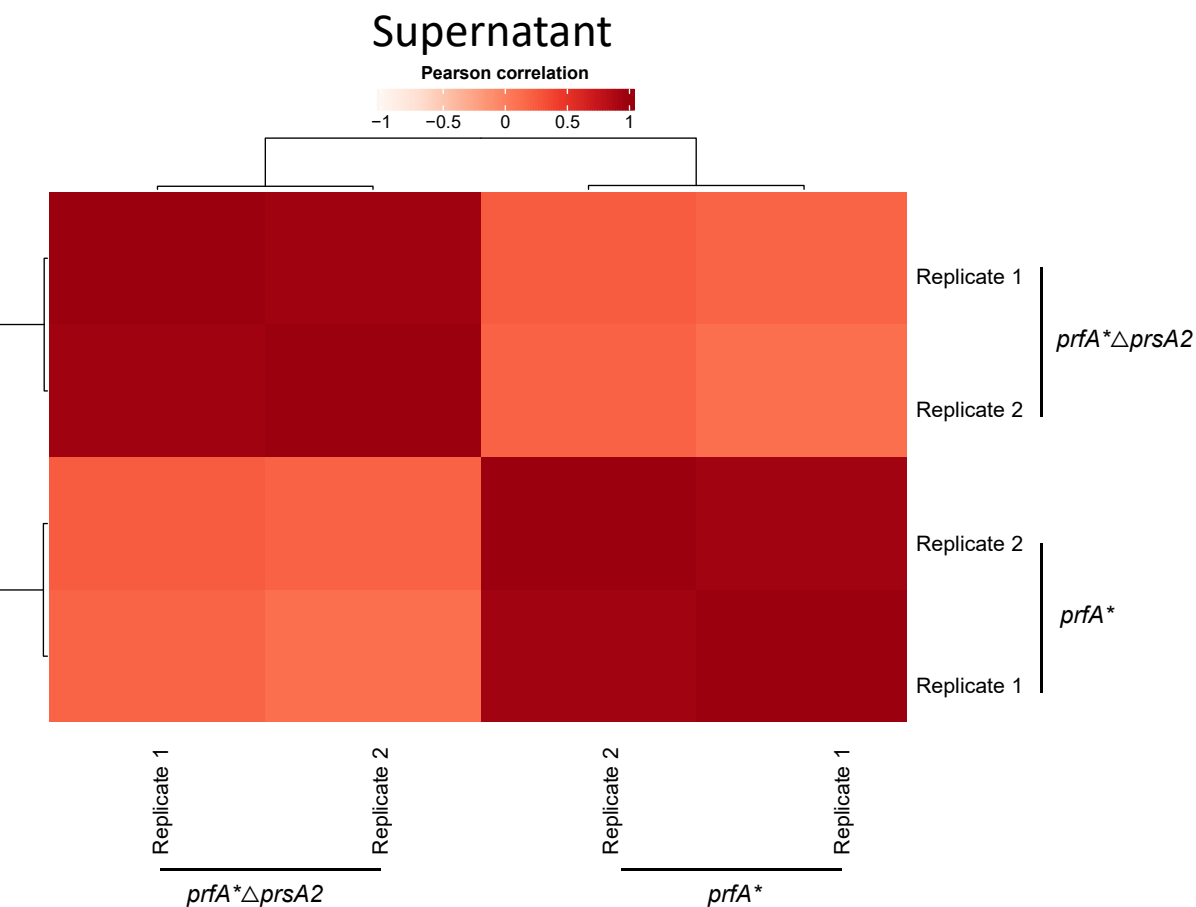

Figure S10

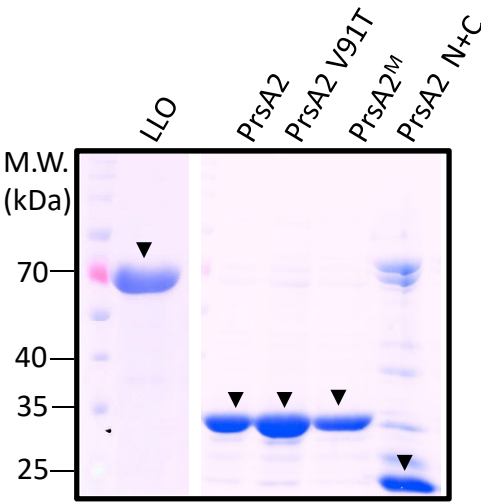

Supplement: Supplemental Figures — Figures S1-S10. [file mbio.00743-24-s0001.pdf]
