# Supplementary material for: The chaperone PrsA2 regulates the secretion, stability, and folding of listeriolysin O during Listeria monocytogenes infection
Source: mBio. 2024 May 29;15(7):e00743-24. doi: 10.1128/mbio.00743-24 (PMC11253611; doi:10.1128/mbio.00743-24)
Supplement: Supplemental material — Supplemental figure legends and tables. [file mbio.00743-24-s0002.pdf]

Supplementary Information List

Figure S1. Interaction between *Lm* PrsA2 and LLO.

Figure S2: Particle-size distribution of LLO molecules.

Figure S3. PrsA2 physically interacts with LLO.

Figure S4. Comparison of the secondary structures of Lm PrsA2 and mutants.

Figure S5. Comparison of protein dimer formation by *Lm* PrsA2 and PrsA2 no-dimer mutant/PrsA2^{M}.

Figure S6. PrsA2 mutants are defective in interacting with LLO.

Figure S7. PrsA2-LLO complex prediction.

Figure S8: Immunoblots of bacterial cell fractions.

Figure S9. Pearson's correlation matrix across samples.

Figure S10. Affinity purification of recombinant proteins.

Table S1. Proteins in cell wall fractions that are significantly different in the *Lm prfA strain compared to the *prfA*ΔprsA2* mutant**

Table S2. Proteins in released fractions that are significantly different in the *Lm prfA strain compared to the *prfA*ΔprsA2* mutant**

Table S3. Size distribution of LLO molecules at physiological pH values and temperatures

Table S5. Tandem Mass Tagged MS analysis of Lm *prfA and *prfA*ΔprsA2* strains**

Table S6. List of oligonucleotides

Supplementary Figure Legends

Figure S1. Interaction between *Lm* PrsA2 and LLO. Immunoblots of the immunoprecipitate (IP) of strains *Lm prfA**, *prfA** Δ*prsA2*, and *prfA** Δ*prsA2* complemented with *prsA2* **(a)** and strains *Lm* wildtype, Δ*prsA2*, and Δ*prsA2* complemented with *prsA2* **(b)** are shown. LLO antibodies were used to IP LLO from *Lm* cell supernatants, and immunoblots of the IP subjected to SDS-PAGE were probed with anti-LLO and anti-PrsA2 antibodies. Bands corresponding to LLO and PrsA2 are arrowed.

Figure S2: Particle-size distribution of LLO molecules. Dynamic light scattering of recombinant LLO was monitored at pH 5.5 (**a**), pH 6.5 (**b**), and pH 7.4 (**c**) at 37°C, collected over 15 minutes. Black arrows show the peaks determined at the different pH levels. **d**, Combination plot of the size distribution of LLO (0-15 minutes, a-c) collected at pH 5.5, 6.5, and 7.4. The blue arrow shows the population of LLO molecules that are partially folded compared to pH 5.5. The folded intermediates of LLO are averaged and labeled as folded; pH 5.5 (17.57 nm), partially folded; pH 6.5 (26. 41 nm), and aggregates; pH 7.4 (694. 71 nm) based on the multiple equilibrium unfolding model(1). The hydrodynamic radius of LLO at pH 6.5 and 7.4 was compared to the hydrodynamic radius of LLO at pH 5.5 using the unpaired, two-tailed Student's *t*-test, **P*<0.05. All experiments were performed in triplicate with similar results.

Figure S3. PrsA2 physically interacts with LLO. **a,** ITC thermogram controls of buffer titrated into PrsA2 at pH of 6.5 (orange) and titration of PrsA2 into buffer at the same pH level (blue). **b,** MST thermogram of PrsA2-NHS titrated into LLO at pH of 6.5 and 37°C. PrsA2-NHS concentration was kept constant at 20 nM while the concentration of LLO was varied from 0.114 nM to 3750 nM. The resulting K_D from the interactions is 28.0 [18, 40] nM. **c,** ITC thermogram of PrsA2 titrated into LLO at pH of 6.5 at 37°C. 64 μM PrsA2 was titrated into 1.8 μM LLO and the fitted curve correspond to n= 2PrsA2:1LLO (1.729 ± 0.058), Kd = 88.32 ± 31.11 nM, Δ*H* = -129.1 ± 6.542 kJ/mol, Ka= 1.132E7M(^-1), Δ*G*=-41.88 kJ/mol, and Δ*S* =-281.1 J/mol.K, -*T*Δ*S* = 85.70 kJ/mol . **d,** MST thermogram of PrsA2 labeled with the NHS dye titrated against unlabeled LLO target at pH of 5.5 and 25°C. The concentration of labelled PrsA2 was kept

constant at 20 nM while the concentration of LLO was varied from 0.259 nM to 8500 nM and the fitted curve gives a K_D of 150.0 [70, 320] nM. **e,** MST thermogram of PrsA2-NHS titrated into LLO at pH of 5.5 and 37°C. PrsA2-NHS concentration was kept constant at 20 nM while the concentration of LLO was varied from 0.259 nM to 8500 nM. The resulting K_D from the interactions is 190.0 [70, 470] nM. **f,** ITC thermogram of PrsA2 titrated into LLO at pH of 5.5 at 25°C. 117 µM PrsA2 was titrated into 3 µM LLO and the fitted curve correspond to n= 2PrsA2:1LLO(1.962 ± 0.059), Kd = 201.0 ± 61.22 nM, ΔH = -33.16 ± 1.496 kJ/mol, Ka= 4.9975E6M(^-1) , ΔG= -38.23 kJ/mol, and ΔS =16.98 J/mol.K, -$T\Delta S$ =-5.062 kJ/mol. **g,** ITC thermogram of PrsA2 titrated into LLO at pH of 5.5 at 37°C. 234 µM PrsA2 was titrated into 7 µM LLO and the fitted curve correspond to n= 2PrsA2:1LLO(1.948 ± 0.063), Kd = 330.30 ± 127.1 nM, ΔH = -22.97 ± 1.132 kJ/mol, Ka= 3.028E6M(^-1), ΔG=-38.48 kJ/mol, and ΔS =50.03 J/mol.K, -$T\Delta S$ =-15.52 kJ/mol. **h,** MST thermogram of PrsA2-NHS titrated into LLO at pH of 7.4 and 25 °C. PrsA2-NHS concentration was kept constant at 20 nM while the concentration of LLO was varied from 0.473 nM to 1550 nM. The resulting K_D from the interactions is 23.0 [14, 33] nM. **i,** MST thermogram of PrsA2-NHS titrated into LLO at pH of 7.4 and 37 °C. PrsA2-NHS concentration was kept constant at 20 nM while the concentration of LLO was from 0.473 nM to 1550 nM. The resulting K_D from the interactions is 39.0 [21, 66] nM. **j,** ITC thermogram of PrsA2 titrated into LLO at pH of 7.4 at 25°C. 40 µM PrsA2 was titrated into 1.0 µM LLO and the fitted curve correspond to n= 2PrsA2:1LLO (2.11 ± 0.046), Kd = 46.10 ± 22.18 nM, ΔH = -90.96 ± 2.98 kJ/mol, Ka= 2.169E7M(^-1) , ΔG=-43.56 kJ/mol, and ΔS =-152.80 J/mol.K, -$T\Delta S$=47.40 kJ/mol. **k,** ITC thermogram of PrsA2 titrated into LLO at pH of 7.4 at 37°C. 70 µM PrsA2 was titrated into 3.6 µM LLO and the fitted curve correspond to n= 2PrsA2:1LLO (2.012 ± 0.029), Kd = 67.23 ± 18.56 nM, ΔH = -53.65 ± 1.255 kJ/mol, Ka= 1.487E7M(^-1), ΔG= -42.59 kJ/mol, and ΔS =-35.68 J/mol.K, -$T\Delta S$= -35.68 kJ/mol. All MST data were analyzed using PALMIST and the figures were rendered using GUSSI v1.2.0. The top panels show the thermophoretic time-traces of three or more experiments with blue and pink areas depicting time-spans used to obtain the fluorescence hot (F_h) and cold (F_c) regions. All the time traces are normalized to a starting value of 1.0. The middle panel shows the binding curve with baseline fluorescence subtracted and the error bars of the non-linear curve represent the standard deviation. The line of best was determined using the 1:1 binding model with 95% confidence using error surface projection as published previously (2, 3). Bottom panels represent the

residuals between the fit and the data plotted. All ITC data were analyzed using an independent binding model in the NanoAnalyze Software v2.2.0 (TA instruments). The error of each of the thermodynamic parameter was determined from the independent fitting model applied to the ITC data using 95% confidence interval. Experiments were carried out three or more times.

Figure S4. Comparison of the secondary structures of *Lm* PrsA2 and mutants. Circular dichroism spectra of PrsA2 and mutants plotted as millidegrees.

Figure S5. Comparison of protein dimer formation by *Lm* PrsA2 and PrsA2 no-dimer mutant/PrsA2^{M}. SDS-PAGE and western analysis of PrsA2 and PrsA2^{M} mutant treated with a chemical crosslinker (+) and without a chemical crosslinker (-).

Figure S6. PrsA2 mutants are defective in interacting with LLO. **a,** MST thermogram of PrsA2V91T-NHS titrated into LLO at pH of 6.5 and 25 ^{o}C. PrsA2V91T-NHS concentration was kept constant at 10/20 nM while the concentration of LLO was varied from 0.381 nM to 1250 nM. The resulting K_D from the interactions is 2.80 [1.2, 5.3] nM. **b,** ITC thermogram of PrsA2V91T titrated into LLO at pH of 6.5 at 25°C. 141.6 µM PrsA2 V91T was titrated into 4.0 µM LLO and the fitted curve correspond to n= 2PrsA2:1LLO (2.115 ± 0.015), Kd = 9.644 ± 4.19 nM, Δ*H* = -17.62 ± 0.272 kJ/mol, Ka= 1.037E8M(^-1) , Δ*G*= -45.75 kJ/mol, and Δ*S* = 94.36 J/mol.K, -*T*Δ*S*= -28.13 kJ/mol. **c,** MST thermogram of PrsA2 N+C-NHS titrated into LLO at pH of 6.5 and 25 ^{o}C. PrsA N+C-NHS concentration was kept constant at 20 nM while the concentration of LLO was varied from 0.595 nM to 1950 nM. The resulting K_D from the interactions is 330.0 [130, 860] nM. **d,** ITC thermogram of PrsA N+C titrated into LLO at pH of 6.5 at 25°C. 160 µM PrsA2 N+C was titrated into 5 µM LLO and the fitted curve correspond to n= 2PrsA2:1LLO (2.080 ± 0.074), Kd = 356.7 ± 128.2 nM, Δ*H* = -69.32 ± 3.210 kJ/mol, Ka= 2.804E6M(^-1), Δ*G*= -36.80 kJ/mol, and Δ*S* =-109.1 J/mol.K, -*T*Δ*S*= 32.51 kJ/mol. **e,** MST thermogram of PrsA2 No dimer (PrsA2^{M})-NHS titrated into LLO at pH of 6.5 and 25°C. PrsA No dimer (PrsA2^{M})-NHS concentration was kept constant at 20 nM while the concentration of LLO was varied from 0.595 nM to 1950 nM. The resulting K_D from the interactions is 190.0 [80, 490] nM. **f,** ITC thermogram of PrsA No dimer (PrsA2^{M}) titrated into LLO at pH of 6.5 at 25°C. 180 µM PrsA2 No dimer (PrsA2^{M}) was titrated into 5.5 µM LLO and the fitted curve correspond to n= 2 PrsA2:1LLO (2.068 ± 0.044), Kd = 206.2 ± 59.08 nM, Δ*H* = -89.30 ± 2.702 kJ/mol, Ka=

4.850E6M(^-1), ΔG= -38.16 kJ/mol, and ΔS =-171.5 J/mol.K, -$T\Delta S$= 51.14 kJ/mol. All MST and ITC data were analyzed as in figure S2.

Figure S7. PrsA2-LLO complex prediction. **a,** Heat map of the predicted aligned error (PAE) of the top-ranked Alphafold2 PrsA2-LLO complex. The PrsA2 (Foldase and PPIase) and LLO (cholesterol binding domain) interacting surfaces are shown with red and blue panels, respectively. PrsA2 monomer 1 and 2 are denoted as PrsA2M1 (purple panel) and PrsA2M2 (cyan panel) respectively and the predicted LLO structure is shown as an orange panel. The heatmap was rendered using the PAE viewer(4).

Figure S8: Immunoblots of bacterial cell fractions. Immunoblots of cell fractions subjected to SDS-PAGE from *Lm* wildtype, Δ*prsA2*, *prfA** and Δ*inlA* strains. BCA assay was used to ensure equivalent protein amounts were loaded. Blots were probed with anti-PrsA2 **(a)** and anti-InlA antibodies **(b)**, respectively. Secreted (SEC), cell wall (CW), and protoplast (PP) fractions are indicated. Bands corresponding to PrsA2 (~32 kDa) and InlA (~80 kDa) are arrowed.

Figure S9. Pearson's correlation matrix across samples. A heat map showing the Pearson's correlations between samples from *Lm prfA** and *prfA**Δ*prsA2* strains for cell wall (**a**) and supernatant fractions (**b**), respectively. Two replicates were performed for each condition, labeled as replicate 1 and 2, respectively.

Figure S10. Affinity purification of recombinant proteins. Ni-NTA affinity purified recombinant proteins were separated by SDS-PAGE and stained with Coomassie blue dye. Recombinant LLO, PrsA2, and PrsA2 mutant bands are arrowed.

Supplementary Tables

Table S1. Proteins in cell wall fractions that are significantly different in the *Lm prfA strain compared to the *prfA*ΔprsA2* mutant**

Functional class	Cellular Localization	Increased/ Decreased	Gene Number	Protein name	Protein description
Bacterial physiology	cw	Increased	lmrg2116	GbuC	Glycine betaine/carnitine transport binding protein
	m	Increased	lmo1547	MreC	Cell shape-determining protein
	c	Increased	lmo2457	TpiA1	Triosephosphate isomerase 1
	c	Increased	lmo2653	Tuf	Elongation factor Tu
	c	Increased	lmo0188	RsmA	Ribosomal RNA small subunit methyltransferase A
	c	Increased	lmo1267	Tig	Trigger factor
	c	Increased	lmo0228	LysS	Lysine--tRNA ligase
	c	Increased	lmo2654	FusA	Elongation factor G
	c	Increased	lmo1011	DapH	2,3,4,5-tetrahydropyridine-2,6-dicarboxylate N-acetyltransferase
	c	Increased	lmo1685	HemL2	Glutamate-1-semialdehyde 2,1-aminomutase 2
	c	Increased	lmo2538	Upp	Uracil phosphoribosyltransferase
	c	Increased	lmo1896	AsnS	Asparagine--tRNA ligase
	c	Increased	lmo1355	Efp	Elongation factor P
	c	Increased	lmo0055	PurA	Adenylosuccinate

					synthetase
	c	Increased	lmo2118	GlmM	Phosphoglucosamine mutase
	c	Decreased	lmo2531	AtpA2	ATP synthase subunit alpha 2
	c	Decreased	lmo0943	Dps	DNA protection during starvation protein
	c	Decreased	lmo1902	PanB	3-methyl-2-oxobutanoate hydroxymethyltransferase
	m	Decreased	lmo1849	MntB	Manganese transport system ATP-binding protein
	c	Decreased	lmo0211	RplY	Large ribosomal subunit protein bL25
	c	Decreased	lmo2529	AtpD2	ATP synthase subunit beta 2
	c	Decreased	lmo2566	GcvH	Lipoyl-[GcvH]:protein N-lipoyltransferase
	c	Decreased	lmo2785	Kat	Catalase
	c	Decreased	lmo1877	Fhs	Formate--tetrahydrofolate ligase
	c	Decreased	lmo1826	RpoZ	DNA-directed RNA polymerase subunit omega
	c	Decreased	lmo2743	Tal1	Probable transaldolase 1
	c	Decreased	lmo0539	LacD	Tagatose 1,6-diphosphate aldolase
	c	Decreased	lmo1349	gcvPA	Probable glycine dehydrogenase (decarboxylating) subunit 1

	c	Decreased	lmo1348	GcvT	Aminomethyltransferase
	c	Decreased	lmo0534	Lmo0534	Hypothetical protein
	c	Decreased	lmo2363	GadB	Glutamate decarboxylase beta
	c	Decreased	lmo2205	GpmA	2,3-bisphosphoglycerate-dependent phosphoglycerate mutase
	m	Decreased	lmo1350	gcvPB	Probable glycine dehydrogenase (decarboxylating) subunit
	c	Decreased	lmo1660	leuS	Leucine--tRNA ligase
	c	Decreased	lmo1504	Alas	Alanine--tRNA ligase
	m	Decreased	lmo1847	MntA	Manganese-binding lipoprotein
	c	Decreased	lmo0251	RpII	Large ribosomal subunit protein bL12
	c	Decreased	lmo1995	DeoC	Deoxyribose-phosphate aldolase
Bacterial Virulence	c	Increased	lmo2213	IsdG	Heme-degrading monooxygenase
	c	Increased	lmo2072	Rex	Redox-sensing transcriptional repressor
	m	Decreased	lmo0204	ActA	Actin assembly-inducing protein
	s	Decreased	lmo0434	InlB	Internalin B
	nc	Decreased	lmo0203	Mpl	Zinc metalloproteinase
	s	Decreased	lmo0433	InlA	Internalin A
	s	Decreased	lmp0202	LLO	Listeriolysin O
	nc	Decreased	lmp0201	PlcA	1-phosphatidylinositol

					phosphodiesterase
	m	Decreased	lmo0205	PlcB	Phospholipase C
	nc	Decreased	lmo0582	Iap	Invasion-associated secreted endopeptidase
	s	Decreased	lmo2219	PrsA2	Foldase protein PrsA2
	nc=non-classically secreted, m=membrane, s=secreted, cw=cell wall. Cellular localization was done using the SignalP-6.0 server.				

Table S2. Proteins in released fractions that are significantly different in the *Lm prfA strain compared to the *prfA*ΔprsA2* mutant**

**Functional class**	**Cellular localization**	**Increased/ Decreased**	**Gene Number**	**Protein name**	**Protein description**
Bacterial physiology	c	Increased	lmo0208	Hypothetical protein	
	c	Increased	lmo1840	PyrR	Bifunctional protein PyrR
	c	Increased	lmo1450	CshB	DEAD-box ATP-dependent RNA helicase
	c	Increased	lmo1885	xpt	Xanthine phosphoribosyltransferase
	c	Increased	lmo1012	lmo1012	N-acetyldiaminopimelate deacetylase
	c	Increased	lmo1896	AsnS	Asparagine--tRNA ligase
	c	Increased	lmo1553	hemL1	Glutamate-1-semialdehyde 2,1-aminomutase 1
	c	Increased	lmo2190	MecA	Adapter protein MecA
	c	Increased	lmo1268	ClpX	ATP-dependent Clp protease ATP-binding subunit ClpX
	c	Increased	lmo2030	SepF	Cell division protein SepF
	c	Decreased	lmo2434	lmo2434	Probable glutamate decarboxylase gamma
	c	Increased	lmo0539	LacD	Tagatose 1,6-diphosphate aldolase
	m	Decreased	lmo1849	MntB	Manganese transport system ATP-binding protein

	s	Decreased	lmo0207	Lmo0207	Uncharacterized lipoprotein
	cw	Decreased	Lmo2034	DivlB	Cell division protein
	m	Decreased	lmo1847	MntA	Manganese-binding lipoprotein
Bacterial Virulence	c	Increased	lmo2213	IsdG	Heme-degrading monooxygenase
	m	Decreased	lmo0204	ActA	Actin assembly-inducing protein
	s	Decreased	lmp0202	LLO	Listeriolysin O
	nc	Decreased	lmo0582	Iap	Invasion-associated endopeptidase p60
	m	Decreased	lmo0205	PlcB	Phospholipase C
	s	Decreased	lmo1388	tcsA	CD4+ T-cell-stimulating antigen
	s	Decreased	lmo2219	PrsA2	Foldase protein PrsA2
	nc=non-classically secreted, m=membrane, s=secreted, cw=cell wall. Cellular localization was done using the SignalP-6.0 server.				

Table S3. Size distribution of LLO molecules at physiological pH values and temperatures

Time (minutes)	Particle size distributions					
	LLO (pH 5.5)		LLO (pH 6.5)		LLO (pH 7.4)	
	Hydrodynamic radius	Log(U/A)	Hydrodynamic radius	Log(U/A)	Hydrodynamic radius	Log(U/A)
0	17.12 ± 3.96	0	23.97 ±12.07*	0.14*	129.52 ±91.66*	0.87*
7	17.27 ± 4.51	0.003	24.24 ±11.78*	0.15*	496.65 ±271.99*	1.46*
15	18.31 ± 5.66	0.02	31.02 ±17.33*	0.25*	1457.97 ±791.08*	1.93*

The plotted hydrodynamic radius was determined as the average of the two or more peaks at the various pH levels. The fraction of LLO aggregates (shown as Log of unfolded or/aggregated LLO molecules, U/A) was determined by assuming that the hydrodynamic radius of LLO aggregates is proportional to fully folded LLO. The smallest radius of LLO is assumed to represent fully folded LLO, and the highest radius corresponds to the LLO in its aggregated form based on the multiple equilibrium unfolding model (Fan *et a*l., 1998). The hydrodynamic radius of LLO at pH 6.5 and 7.4 was compared to the hydrodynamic radius of LLO at pH 5.5 using the unpaired, two-tailed Student's *t*-test, *P<0.05. Data were represented as mean ± standard deviation, and all experiments were performed at least three independent times. U and A denotes unfolded LLO and LLO aggregates, respectively.

Table S4. List of bacterial strains and plasmids

Strain/Plasmid	Genotype	Designation	Source/reference
TOP10	*E. coli* host strain used for recombinant pPL2 plasmids		Invitrogen
SM10	*E. coli* host strain for conjugation of pPL2 plasmids		
LAC-153	*E. coli* BL21 (DE3*) for PrsA2, PrsA2 mutants and LLO protein expression and purification	DE3*	Invitrogen
NF-L100	*L. monocytogenes* 10403S parent strain		(5)
NF-L1167	10403S actA-gus-neo *prfA*(L140F)	*prfA* [prfA(L140F)]*	(6)
NF-L1637	NF-L1167 [*prfA*(L140F) with Δ*prsA2*::erm]	*prfA*ΔprsA2*	(7)
NF-L1659	NF-L1637 [*prfA*(L140F) Δ*prsA2*::erm] with integrated pPL2-*prsA2* (pNF1255)	*prfA*ΔprsA2+ pPL2-prsA2*	(7)
NF-L1651	10403S with Δ*prsA2*::erm	Δ*prsA2*	(7)
NF-L1656	NF-L1651 (Δ*prsA2*::erm) with integrated pPL2-*prsA2* (pNF1255)	Δ*prsA2+ pPL2-prsA2*	(7)
NF-L3804	10403S with ΔprsA2::erm with integrated pPL2-*prsA2* (V91T)	Δ*prsA2+ pPL2-prsA2* (V91T)	(8)
NF-L1674	10403S with Δ*prsA2*::erm with integrated pPL2-*prsA2* (N+C)	Δ*prsA2+ pPL2-prsA2* (N+C)	(9)
NF-L3806	10403S with Δ*prsA2*::erm with integrated pPL2-*prsA2 (No dimer mutant)/ prsA2^M*	Δ*prsA2+ pPL2-prsA2 (No dimer)/prsA2^M*	(8)
NF-E1764	pQE60 -*prsA2* (amino acids 21-293)	PrsA2-6-his	(9)
NF-E1942	pQE60 -*prsA2* (N+C) (starting at amino acid 21)	PrsA2 N+C	(9)
LAC-150	pQE60 -prsA2 (V91T) (amino acids 21-293)	PrsA2 V91T	This work
LAC-151	pQE60 -*prsA2* (No dimer mutant)/ prsA2^M (V28A/L40A/Y41A/M44A) (amino acids 21-293)	PrsA2 (No dimer mutant) /PrsA2^M	This work
LAC-152	pQE30 -LLO (amino acids 25-529)	LLO-6-His	This work

Table S5. Tandem Mass Tagged MS analysis of Lm *prfA** and *prfA*ΔprsA2* strains

			*prfA**				*prfA** Δ*prsA*2			
			**Cell Wall**		**Released**		**Cell Wall**		**Released**	
**Protein**	**Mol. Weight**	**Localization**	**Log2 Fold Change**	**p-value**	**Log2 Fold Change**	**p-value**	**Log2 Fold Change**	**p-value**	**Log2 Fold Change**	**p-value**
AccA	35 kDa	Cytoplasmic	Not detected	Not detected	-0.388087966	0.066609568	Not detected	Not detected	-0.166567636	0.011129994
AccD	32 kDa	Cytoplasmic	-0.385179059	*	-0.253889098	0.122799334	0.689286571	*	-0.042922771	0.683295865
AckA1	44 kDa	Cytoplasmic	-0.597333761	0.013513177	-0.316294432	0.088113211	0.57916284	0.011935637	-0.006742632	0.912311592
ActA	70 kDa	Membrane	3.163283615	0.008493375	3.755435167	0.018177368	-1.062098856	0.018946686	-1.726083459	0.031098779
AcyP	10 kDa	Cytoplasmic	-0.613307856	0.014576102	-0.561047029	0.011687507	0.441747091	0.231558347	0.076816568	0.355110145
AddB	133 kDa	Cytoplasmic	Not detected	Not detected	-0.514138584	*	Not detected	Not detected	0.034497073	*
Ade	62 kDa	Cytoplasmic	Not detected	Not detected	-0.358426702	0.021115634	Not detected	Not detected	-0.013799965	0.835657297
Adk	24 kDa	Cytoplasmic	-0.231979045	0.483391438	-0.309683901	0.871375673	0.839221909	0.170801192	-0.072807761	0.9268131
AlaS	98 kDa	Cytoplasmic	-0.452044359	0.392079792	-0.415815171	0.392348535	-0.202975262	0.608348309	0.060075051	0.912612906
Apt	19 kDa	Cytoplasmic	Not detected	Not detected	-0.258754761	0.078308627	Not detected	Not detected	-0.111717476	0.034931149
ArgG	44 kDa	Cytoplasmic	Not detected	Not detected	-0.393237333	0.001463389	Not detected	Not detected	-0.197505907	0.068895743
ArgR	17 kDa	Cytoplasmic	Not detected	Not detected	-0.381929436	0.097504903	Not detected	Not detected	-0.046410947	0.771561674
ArgS	63 kDa	Cytoplasmic	-0.663319082	0.031803543	-0.158846177	0.144194429	0.261177567	0.457819638	0.07847212	0.384296627
AroA	46 kDa	Cytoplasmic	Not detected	Not detected	-0.449347986	0.020091048	Not detected	Not detected	-0.026864676	0.799369433
AroB	41 kDa	Cytoplasmic	Not detected	Not detected	-0.634238584	*	Not detected	Not detected	-0.028502927	*
AroC	42 kDa	Cytoplasmic	-0.5466898	0.096229165	-0.4978865	0.098341436	0.977040744	0.100907558	-0.0090010	0.593797874

			84		67				93	
AroD	28 kDa	Cytoplasmic	Not detected	Not detected	-0.707857566	0.150082015	Not detected	Not detected	0.347784611	0.460663999
AsnS	49 kDa	Cytoplasmic	-0.75485459	0.012300857	-0.727221251	0.003975137	1.100538278	0.044814687	0.306659343	0.032684535
AspS	66 kDa	Cytoplasmic	-0.837715687	0.024934273	-0.534879388	0.097839422	0.833935944	0.090087482	-0.248576062	0.196040051
AtpA2	55 kDa	Cytoplasmic	0.30710412	0.023136496	0.092421197	0.786604945	-0.488819497	0.004407731	-0.024231998	0.941689416
AtpD2	52 kDa	Cytoplasmic	0.350525471	0.175036876	-0.232826746	0.191275107	-0.172668489	0.384117875	-0.083949299	0.485661595
AzoR1	23 kDa	Cytoplasmic	Not detected	Not detected	-0.322459059	0.075829429	Not detected	Not detected	0.093779874	0.278071238
AzoR2	23 kDa	Cytoplasmic	-0.714351984	0.002162774	-0.491898045	0.061370053	0.964251669	0.120236063	-0.103814129	0.558194234
Buk	39 kDa	Cytoplasmic	Not detected	Not detected	-0.088370791	0.741364572	Not detected	Not detected	0.14564414	0.682855549
CheA	69 kDa	Cytoplasmic	Not detected	Not detected	-0.332138584	*	Not detected	Not detected	0.189397073	*
CheY	13 kDa	Cytoplasmic	Not detected	Not detected	-0.654641074	0.160321676	Not detected	Not detected	-0.013997252	0.977462614
CinA	46 kDa	Cytoplasmic	Not detected	Not detected	-0.387564132	0.00188265	Not detected	Not detected	-0.076781737	0.080365951
ClpB	98 kDa	Cytoplasmic	-0.496224907	0.107501707	-0.452931247	0.021715184	0.655449266	0.019075503	-0.008480437	0.893922043
ClpP	22 kDa	Cytoplasmic	-0.328351984	0.049209748	-0.433237461	0.177292415	0.142609202	0.552006247	0.120790988	0.559430586
ClpX	46 kDa	Cytoplasmic	-0.77349995	0.165515969	-0.610337624	0.011619715	0.293682036	0.41224049	0.22186074	0.091341348
Cmk	25 kDa	Cytoplasmic	-0.379764174	0.461647907	-0.415767831	0.104988319	0.837489118	0.267230152	-0.042091798	0.583567459
CobB	26 kDa	Cytoplasmic	Not detected	Not detected	-0.567038584	*	Not detected	Not detected	-0.128802927	*
CodY	29 kDa	Cytoplasmic	-0.603281294	0.008284246	-0.747594874	0.072311106	0.525744108	0.021087432	0.333140188	0.368364821
CshA	57 kDa	Cytoplasmic	-0.42730864	0.222604717	-0.609043356	0.028030458	0.322126605	0.289898753	-0.118231688	0.453248805

CshB	50 kDa	Cytoplasmic	Not detected	Not detected	-0.762566215	0.010243276	Not detected	Not detected	0.286845233	0.159726298
CspLA	7 kDa	Cytoplasmic	-0.375153733	0.629763943	-0.461264219	0.159356283	0.61205903	0.454717414	-0.087852094	0.755919641
CspLB	7 kDa	Cytoplasmic	-0.336154116	0.211501845	-0.681411298	0.216228655	0.294068286	0.21217212	-0.273995813	0.560170762
CysS	54 kDa	Cytoplasmic	-0.64610801	0.080585373	-0.405053582	0.109067463	-0.019998068	0.858718635	-0.078109146	0.159205375
DapA	31 kDa	Cytoplasmic	-0.346420602	0.302893692	-0.416265441	0.235770328	0.108462925	0.738423207	0.268351832	0.506449331
DapB	29 kDa	Cytoplasmic	-0.628479059	*	-0.350287678	0.135403878	1.546986571	*	-0.110209297	0.332729303
DapF	36 kDa	Cytoplasmic	-0.517279059	*	-0.27943858	0.029845593	0.516053238	*	-0.113382399	0.506067286
DapH	25 kDa	Cytoplasmic	-0.69398974	0.004196518	-0.347790327	0.004282303	1.308649344	0.009268362	0.033774218	0.047407588
Dat	32 kDa	Cytoplasmic	-0.149079059	*	-0.279460771	0.112005039	0.851486571	*	-0.091070452	0.329590511
Ddl	41 kDa	Cytoplasmic	-0.585411117	0.015237972	-0.486838223	0.051065495	0.899087855	0.005877542	0.005940441	0.941895321
Def	21 kDa	Cytoplasmic	Not detected	Not detected	-0.345487843	0.063989531	Not detected	Not detected	-0.126652624	0.162680794
DeoB	44 kDa	Cytoplasmic	-0.605700808	0.05514751	-0.343421476	0.213125296	0.730873753	0.061452062	0.103789391	0.666426049
DeoC	24 kDa	Cytoplasmic	0.079072201	0.86612939	-0.417801512	0.110985024	-0.178627999	0.670431434	-0.264684764	0.348128029
DeoD	25 kDa	Cytoplasmic	-0.653836178	0.045439321	-0.339415537	0.062583721	0.244487363	0.255135007	0.031322183	0.806441097
Der	49 kDa	Membrane	-0.934641507	0.125476666	-0.502945497	0.00169204	0.892847056	0.024480996	-0.020052179	0.484541809
DivIB	30 kDa	Cell Wall	-0.389202051	0.166153923	0.340090995	0.019581568	-0.028002961	0.923114807	-0.4463245	0.011683978
DltA	58 kDa	Cytoplasmic	-0.446184703	0.14894876	-0.337262409	0.216926817	0.591042101	0.204603156	0.320550453	0.302140394
DltC	9 kDa	Cytoplasmic	Not detected	Not detected	-0.671587154	0.29419849	Not detected	Not detected	0.438115997	0.274494586
DnaA	51	Cytoplasmic	Not	Not	-	0.2861602	Not	Not	0.0566075	0.8612554

	kDa		detected	detected	0.471597757	76	detected	detected	05	06
DnaJ	41 kDa	Cytoplasmic	-0.412879179	0.055439157	-0.580145603	0.071337862	0.277950502	0.197693562	0.271161031	0.269745984
DnaK	66 kDa	Cytoplasmic	-0.583991293	0.081230877	-0.32117896	0.277360015	0.511765063	0.138340007	-0.052826576	0.823941621
Dps	18 kDa	Cytoplasmic	-0.507246241	0.026685795	-0.630858823	0.119903746	-0.703943693	0.033652294	-0.183322886	0.038545006
Dxs	67 kDa	Cytoplasmic	Not detected	Not detected	-0.567052879	0.015009716	Not detected	Not detected	0.095018545	0.153283074
Efp	20 kDa	Cytoplasmic	-0.371489749	0.412377864	-0.377653657	0.057951468	1.289154412	0.031497144	-0.074840133	0.28997453
EngB	23 kDa	Cytoplasmic	Not detected	Not detected	-0.566031843	0.021312989	Not detected	Not detected	0.069271863	0.728016549
Eno	46 kDa	Cytoplasmic	-0.624292551	0.177502177	-0.631401643	0.135738258	0.181101072	0.485217599	-0.063886866	0.860952597
Era	35 kDa	Membrane	-0.61900801	0.064572333	-0.514394746	0.004042427	0.945733672	0.145034185	0.004871623	0.955899202
EzrA	67 kDa	Membrane	-1.038379059	*	-0.163838584	*	0.641286571	*	0.351997073	*
FabH	34 kDa	Cytoplasmic	-0.331451607	0.202885965	-0.428402538	0.025051811	0.471439029	0.069004167	-0.111224949	0.011172019
FapR	21 kDa	Cytoplasmic	Not detected	Not detected	-0.341289506	0.01221967	Not detected	Not detected	-0.010458881	0.94698131
Fhs	60 kDa	Cytoplasmic	-0.760383072	0.164628117	-0.554315822	0.169096157	-0.30387166	0.185646714	0.252384218	0.479253672
FlaA	30 kDa	Cytoplasmic	-1.378099969	0.031333429	-0.577598286	0.300940788	0.216701599	0.674468676	-0.436012832	0.199173912
FliE	10 kDa	Cytoplasmic	-1.154003058	0.412825397	-0.345767227	0.24363891	-0.017203976	0.989737895	-0.00062325	0.984950498
Fmt	34 kDa	Cytoplasmic	Not detected	Not detected	Not detected	Not detected	Not detected	Not detected	Not detected	Not detected
Fni	39 kDa	Cytoplasmic	Not detected	Not detected	-0.298103931	0.035635855	Not detected	Not detected	0.164990988	0.186115666
FolD	31 kDa	Cytoplasmic	-0.501527042	0.03272656	-0.440905251	*	0.276406291	0.205076777	-0.33213626	*
FolE	21 kDa	Cytoplasmic	Not detected	Not detected	-0.331826173	0.236646014	Not detected	Not detected	0.176980926	0.389214831

Frr	21 kDa	Cytoplasmic	-0.656244114	0.187451506	-0.449813505	0.100190059	0.583674988	0.204566427	-0.116853677	0.597317822
FtsK	84 kDa	Membrane	Not detected	Not detected	-0.00739889	0.890056	Not detected	Not detected	-0.038088804	0.701143304
FumC	50 kDa	Cytoplasmic	-0.579692272	0.067908823	-0.462287354	0.210285479	0.274224464	0.712842176	-0.064357719	0.23090766
FusA	77 kDa	Cytoplasmic	-0.763633937	0.061719681	-0.358683551	0.039155544	1.04766008	0.040398122	-0.071520735	0.136003462
GadB	54 kDa	Cytoplasmic	-0.70675056	0.163309508	-0.840682974	0.067534011	-0.456578972	0.291011659	0.117059318	0.785224447
GatA	52 kDa	Cytoplasmic	-0.637679059	*	-0.423613528	0.029731597	0.463486571	*	0.173403567	0.234390517
GatB	53 kDa	Cytoplasmic	-0.66588698	0.023084789	-0.399824766	0.017778029	0.910847682	0.056463697	0.166452778	0.108179823
GatC	11 kDa	Cytoplasmic	-1.513581796	0.12077419	-0.440012429	0.093729943	0.39716701	0.577290304	0.22472226	0.071253488
GbuA	44 kDa	Membrane	Not detected	Not detected	0.019492255	0.934061957	Not detected	Not detected	-0.060035191	0.887206295
GbuC	33 kDa	Cell Wall	-0.611375254	0.072624405	-0.293572322	0.010937423	1.104546692	0.046278123	0.006524445	0.975547322
GcvH	14 kDa	Cytoplasmic	-0.303863725	0.337336598	-0.513752847	0.014721601	-0.438581802	0.123654441	-0.066948064	0.657034526
GcvPA	49 kDa	Cytoplasmic	-0.706100583	0.184946482	-0.350258544	0.009017769	-0.516107884	0.127988057	-0.19926752	0.271685783
GcvPB	54 kDa	Membrane	-0.394458422	0.490165926	-0.398850441	0.036808723	-0.831413421	0.168383284	-0.177199922	0.455060298
GcvT	40 kDa	Cytoplasmic	-0.111346898	0.677913887	-0.550238584	*	-0.45510201	0.333652994	-0.305402927	*
GlmM	48 kDa	Cytoplasmic	-0.709916094	0.049890461	-0.45464799	0.019836346	1.172989106	0.010489205	0.054381351	0.521498866
GlmS	66 kDa	Cytoplasmic	-0.636394662	0.013489684	-0.455030599	0.039503135	0.68459139	0.00872687	-0.108120469	0.430548699
GlmU	50 kDa	Cytoplasmic	-0.486479059	*	-0.69083087	0.156700881	0.022386571	*	0.024251393	0.985954682
GlpK	55 kDa	Cytoplasmic	-0.829503147	0.018043773	-0.390738584	*	0.399345882	0.054200672	0.094697073	*
GltX	56	Cytoplasmic	-	0.2056950	-	0.0170234	0.2754383	0.1933588	-	0.4658959

	kDa		0.269422601	86	0.408976531	87	57	87	0.09235199	95
GlyA	45 kDa	Cytoplasmic	-0.590720527	0.03900431	-0.389781721	0.071595746	0.426954964	0.014335441	0.010917358	0.944458892
GlyQ	34 kDa	Cytoplasmic	Not detected	Not detected	-0.506387522	0.013948642	Not detected	Not detected	0.132681911	0.091929901
GlyS	79 kDa	Cytoplasmic	-0.893592527	0.038323515	-0.459342722	0.30891982	0.532151618	0.101918925	0.073355749	0.852672072
Gmk	23 kDa	Cytoplasmic	-0.186423984	0.698652298	-0.332687202	0.083254414	0.785663245	0.172900146	-0.091243649	0.382243952
GpmA	26 kDa	Cytoplasmic	-0.460613132	0.225619156	-0.527881554	0.075875066	-0.415566299	0.374279964	-0.151837136	0.612681479
GpmI	56 kDa	Cytoplasmic	-0.460753953	0.214204518	-0.431246267	0.136281827	0.990490641	0.039767282	-0.306729729	0.288604171
GpsA	36 kDa	Cytoplasmic	-0.728679059	*	-0.544467768	0.004948467	1.375086571	*	-0.086399378	0.088271618
GpsB	13 kDa	Cytoplasmic	-0.749180254	0.042803535	-0.109525763	0.403910537	0.228088947	0.539721942	-0.237665954	0.113730993
GreA	17 kDa	Cytoplasmic	-0.422969667	0.409286656	-0.356710326	0.020960275	0.842516784	0.047120314	-0.058733045	0.101088887
GroL	57 kDa	Cytoplasmic	-0.559424622	0.192997547	-0.520239316	0.113603294	0.133300622	0.925982504	0.029930806	0.922127508
GroS	10 kDa	Cytoplasmic	-0.542163297	0.27821409	-0.663874918	0.036802398	0.361791156	0.006006296	-0.057004677	0.493587681
GrpE	22 kDa	Cytoplasmic	-0.741117083	0.058571682	-0.452934827	0.41047936	0.658127438	0.016274389	0.089604775	0.874070287
GshAB	88 kDa	Cytoplasmic	-0.665579059	*	Not detected	Not detected	0.466686571	*	Not detected	Not detected
GuaA	58 kDa	Cytoplasmic	0.565842763	0.960307922	-0.085182974	0.785605867	1.218446875	0.166802369	0.053291716	0.883941948
HemC	34 kDa	Cytoplasmic	Not detected	Not detected	-0.427358823	0.403486666	Not detected	Not detected	0.068165367	0.889190743
HemE	40 kDa	Cytoplasmic	0.176427953	0.976641134	-0.48801966	0.016815453	1.093145644	0.140719998	0.001216052	0.968400617
HemH	35 kDa	Cytoplasmic	-0.584479059	*	-0.428938528	0.009928906	1.020286571	*	-0.045809012	0.448656405
HemL1	47 kDa	Cytoplasmic	-0.6775790	*	-0.6091016	0.021196571	1.583486571	*	0.26849127	0.125935217

			59		96					
HemL2	46 kDa	Cytoplasmic	-0.915437524	0.053532574	-0.462745884	0.049086231	1.505210198	0.025284152	0.034852706	0.826837918
HisC	40 kDa	Cytoplasmic	Not detected	Not detected	-0.092726923	0.791110148	Not detected	Not detected	-0.419370988	0.417110172
HisS	48 kDa	Cytoplasmic	-0.893153188	0.125947089	-0.456023864	0.038746602	0.361028177	0.552476191	-0.1055387 6	0.518760108
Hly	59 kDa	Non-classical	2.455856918	0.002595124	3.54456732	0.00290353	-1.180752385	0.017246937	-1.444189524	0.007153359
Hpf	22 kDa	Cytoplasmic	-0.748386434	0.00813239	-0.499599069	0.08868963	0.025902011	0.718970022	0.011308116	0.970145459
HprK	35 kDa	Cytoplasmic	-0.208179059	*	-0.452504456	0.004872945	-0.105913429	*	0.077040801	0.348393952
HslO	32 kDa	Cytoplasmic	-0.801991735	0.037991841	-0.450708907	0.007725546	0.978547147	0.093351864	0.035431726	0.418650957
HslU	53 kDa	Cytoplasmic	Not detected	Not detected	-0.387070456	0.147930337	Not detected	Not detected	0.173432506	0.509200819
HslV	19 kDa	Cytoplasmic	-0.916402446	0.033144118	-0.469234577	0.064758683	0.168791595	0.536340767	0.097947144	0.517728109
Iap	51 kDa	Non-classical	-0.559928989	0.016043297	0.182753882	0.256912047	-0.330115389	0.104217145	-0.410809297	0.019997711
IleS	104 kDa	Cytoplasmic	-0.862904895	0.043332689	-0.636386464	0.085637576	0.483186874	0.052733257	-0.031725281	0.810936925
InfA	8 kDa	Cytoplasmic	-0.493488964	0.13145544	-0.50220323	0.000305001	0.924287173	0.002263837	-0.080550093	0.075877494
InfB	85 kDa	Cytoplasmic	Not detected	Not detected	0.396261416	*	Not detected	Not detected	0.886197073	*
InfC	20 kDa	Cytoplasmic	-0.410917758	0.126510753	-0.400161034	0.142672035	0.751944702	0.123394507	-0.099252614	0.653346206
InlA	86 kDa	Cell Wall	0.497505218	0.230492829	Not detected	Not detected	-0.617205224	0.123243271	Not detected	Not detected
InlB	71 kDa	Released	1.578649202	0.001185603	Not detected	Not detected	-0.794774662	0.00313435	Not detected	Not detected
IsdG	14 kDa	Cytoplasmic	-0.29049127	0.533766992	-0.410533212	0.103048569	1.259413446	0.054366146	0.367355338	0.043438839
IspE	32 kDa	Cytoplasmic	Not detected	Not detected	-0.102656951	0.728706064	Not detected	Not detected	-0.64776637	0.269352352

IspF	17 kDa	Cytoplasmic	Not detected	Not detected	-0.363840192	0.13075348	Not detected	Not detected	-0.052539942	0.675889384
Kat	56 kDa	Cytoplasmic	-0.498479003	0.09297418	-0.474413153	0.003491769	-0.474522456	0.069112018	0.014156928	0.814303808
LacD	38 kDa	Cytoplasmic	-0.42741419	0.211147651	-0.391410507	0.095697489	-0.452826897	0.217735829	-0.272449572	0.260948843
Ldh1	34 kDa	Cytoplasmic	-0.746643192	0.146440368	-0.584674577	0.025952869	0.151282463	0.661460424	-0.085336926	0.505374588
Ldh2	34 kDa	Cytoplasmic	-0.836979059	*	-0.459000353	0.155968466	1.207186571	*	0.201576727	0.539550411
LepA	68 kDa	Membrane	Not detected	Not detected	-0.580667945	0.017274777	Not detected	Not detected	0.152748947	0.174360794
LeuS	92 kDa	Cytoplasmic	2.740177864	0.220773664	-0.391070756	0.028593677	-0.990844813	0.33144152	-0.052790675	0.690145387
LexA	23 kDa	Cytoplasmic	Not detected	Not detected	-0.596795688	0.070701992	Not detected	Not detected	0.034697644	0.963118396
LipL	31 kDa	Cytoplasmic	Not detected	Not detected	-0.361080052	0.042674509	Not detected	Not detected	-0.104647894	0.353708734
lmo0164	15 kDa	Cytoplasmic	Not detected	Not detected	-0.486719568	0.122591616	Not detected	Not detected	0.161190996	0.568517786
lmo0191	27 kDa	Cytoplasmic	-0.335213045	0.310677075	-0.522962653	0.001462355	0.273778681	0.180372705	-0.007054673	0.905306653
lmo0207	17 kDa	Lipid-anchored	Not detected	Not detected	2.28199704	0.002355094	Not detected	Not detected	-1.393042698	0.003493072
lmo0208	12 kDa	Cytoplasmic	-0.853267165	0.003467494	-1.142755528	0.083800505	0.096171807	0.413206077	0.851439579	0.234447682
lmo0216	10 kDa	Cytoplasmic	Not detected	Not detected	-0.455480575	0.667721879	Not detected	Not detected	0.002779352	0.890232633
lmo0369	27 kDa	Cytoplasmic	-0.764253711	0.142133245	-0.403103717	0.024753425	1.022299462	0.032446496	0.186376861	0.01667178
lmo0392	34 kDa	Membrane	-0.223979059	*	-0.453378533	0.098492499	-0.550013429	*	-0.183102325	0.479717783
lmo0533	10 kDa	Cytoplasmic	-0.248512995	0.511862019	-0.674813525	0.102250204	0.139020372	0.713183727	0.444631726	0.305233394
lmo0534	47 kDa	Cytoplasmic	-0.38915972	0.299502345	-0.479234806	0.044283787	-0.27078293	0.410555752	0.08911864	0.467782232
lmo09	20	Cytoplasmic	-	0.2785987	-	0.0462515	0.9101533	0.1311130	-	0.2057408

35	kDa		0.665377636	06	0.252437905	45	08	04	0.111246063	84
lmo1012	42 kDa	Cytoplasmic	-0.445334406	0.171816022	-0.425037836	0.025036005	0.313683977	0.289999617	0.227691278	0.204771396
lmo1028	8 kDa	Cytoplasmic	-0.384959493	0.378036953	-0.295027324	0.248007218	0.667443506	0.042602325	-0.063187487	0.232141293
lmo1058	10 kDa	Cytoplasmic	-0.870194331	0.033031769	-0.542688562	0.007134298	-0.04795415	0.799858334	-0.031931609	0.610898561
lmo1065	24 kDa	Cytoplasmic	-0.298279059	*	-0.652536846	0.062256612	0.156886571	*	0.054363478	0.749289228
lmo1239	22 kDa	Cytoplasmic	-0.344079059	5.99453E-29	-0.35293276	0.376829161	1.009086571	*	-0.21918105	0.580078684
lmo1304	9 kDa	Cytoplasmic	Not detected	Not detected	-0.901204457	0.016704134	Not detected	Not detected	-0.065836766	0.552259858
lmo1452	41 kDa	Cytoplasmic	Not detected	Not detected	-0.290117413	0.204443874	Not detected	Not detected	-0.000250289	0.980207226
lmo1457	30 kDa	Cytoplasmic	Not detected	Not detected	-0.355529126	0.085266268	Not detected	Not detected	-0.048209307	0.697215314
lmo1501	12 kDa	Cytoplasmic	-1.024913074	0.174001838	-0.513535522	0.165826327	0.777578456	0.074104213	-0.017424426	0.918193706
lmo1502	15 kDa	Cytoplasmic	Not detected	Not detected	-0.467423637	0.348100279	Not detected	Not detected	0.037860265	0.920849304
lmo1503	11 kDa	Cytoplasmic	Not detected	Not detected	-0.238280764	0.450084016	Not detected	Not detected	0.094418545	0.763419379
lmo1577	25 kDa	Cytoplasmic	-0.638488358	0.085268655	-0.437773724	0.08275326	0.012142061	0.929398897	-0.07586374	0.639092679
lmo1608	30 kDa	Cytoplasmic	-0.398624037	0.296215868	-0.405476126	0.21476297	0.195583834	0.639673158	-0.009824649	0.975705033
lmo1687	21 kDa	Cytoplasmic	Not detected	Not detected	-0.249918552	0.071122513	Not detected	Not detected	-0.176910814	0.178115097
lmo1703	51 kDa	Cytoplasmic	Not detected	Not detected	-0.678188786	0.079211095	Not detected	Not detected	0.225267672	0.593601606
lmo1707	9 kDa	Cytoplasmic	Not detected	Not detected	-0.406737843	0.222921679	Not detected	Not detected	0.101806879	0.734470962
lmo1751	51 kDa	Cytoplasmic	Not detected	Not detected	-0.394740522	0.134607525	Not detected	Not detected	-0.081181244	0.691164581
lmo1863	31 kDa	Cytoplasmic	-0.4208790	*	-0.4637105	0.029342739	0.531586571	*	-0.0807942	0.355031082

			59		12				62	
lmo1866	30 kDa	Cytoplasmic	-0.993085111	0.311804909	-0.633950987	0.072817107	-0.766209995	0.405049983	-0.678235794	0.054941071
lmo2046	33 kDa	Cytoplasmic	Not detected	Not detected	-0.717533839	0.212186294	Not detected	Not detected	0.083289284	0.845705868
lmo2113	29 kDa	Cytoplasmic	-0.36917538	0.149448194	-0.631012158	0.011251835	0.220596076	0.221882735	0.156485217	0.132568923
lmo2223	14 kDa	Cytoplasmic	-0.575433158	0.182181292	-0.200109468	0.250879651	0.32305903	0.151647585	-0.124678433	0.345169851
lmo2390	36 kDa	Cytoplasmic	Not detected	Not detected	-0.528966636	0.017638667	Not detected	Not detected	0.109215954	0.2444701
lmo2434	54 kDa	Cytoplasmic	Not detected	Not detected	-0.508172852	0.015396896	Not detected	Not detected	-0.401847519	0.03988581
lmo2473	35 kDa	Cytoplasmic	Not detected	Not detected	-0.426462352	0.002335537	Not detected	Not detected	-0.096501093	0.013356377
lmo2474	34 kDa	Cytoplasmic	-0.538985951	0.066123921	-0.360770642	0.025845837	0.544288369	0.021138153	-0.020250494	0.801450164
lmo2514	31 kDa	Cytoplasmic	Not detected	Not detected	-0.537050355	0.0928079	Not detected	Not detected	0.112526256	0.474525873
lmo2564	7 kDa	Cytoplasmic	-0.289404488	0.137421232	-0.328839193	0.064444051	0.284126444	0.089054459	-0.103668959	0.508017608
lmo2657	53 kDa	Cytoplasmic	7.38128E-05	0.915744878	-0.348104897	0.053999066	0.036518253	0.870803697	0.030768572	0.634642223
lmo2703	11 kDa	Cytoplasmic	Not detected	Not detected	-0.179063784	0.243879832	Not detected	Not detected	-0.11758418	0.633645616
lmo2759	19 kDa	Cytoplasmic	-0.432679059	*	-0.545430258	0.016409898	1.184586571	*	-0.041619655	0.617280845
LuxS	17 kDa	Cytoplasmic	-0.380579059	*	-0.584054915	0.004387015	0.267786571	*	0.145408497	0.298828358
LysS	57 kDa	Cytoplasmic	-0.62174217	0.029449165	-0.440550457	0.047248045	1.154368013	0.072641885	0.019215512	0.717872589
MecA	25 kDa	Cytoplasmic	Not detected	Not detected	-0.559082615	0.067775406	Not detected	Not detected	0.371522764	0.175840338
MenD	65 kDa	Cytoplasmic	-0.384161202	0.145965607	-0.429605088	0.262697722	0.272971482	0.313208979	0.267343375	0.430378109
MenE	52 kDa	Cytoplasmic	Not detected	Not detected	-0.454165974	0.009477929	Not detected	Not detected	-0.154205145	0.019734532

MetE	86 kDa	Cytoplasmic	-0.455079059	*	Not detected	Not detected	-0.426813429	*	Not detected	Not detected
MetG	76 kDa	Cytoplasmic	-0.44345638	0.398663823	-0.390833157	0.052725492	0.794356459	0.069958641	0.044382078	0.787087631
MetK	44 kDa	Cytoplasmic	Not detected	Not detected	-0.426365397	0.027725697	Not detected	Not detected	-0.040225918	0.727073143
MgsA	15 kDa	Cytoplasmic	Not detected	Not detected	-0.079538584	*	Not detected	Not detected	-0.276502927	*
MinC	25 kDa	Cytoplasmic	-0.356927095	0.272197881	-0.341215774	0.078675682	0.699734596	0.074388793	0.14606294	0.102864119
MnmA	41 kDa	Cytoplasmic	-0.353679059	*	-0.439538584	*	0.393586571	*	0.140697073	*
MnmE	50 kDa	Cytoplasmic	Not detected	Not detected	-0.504206282	0.200133941	Not detected	Not detected	0.11591417	0.610324745
MnmG	70 kDa	Cytoplasmic	-0.61589029	0.075467656	-0.370971914	0.046598399	0.254910617	0.20435264	0.04910527	0.179683363
MntA	34 kDa	Membrane	-0.453094136	0.208882229	-0.098183722	0.104288888	-0.344630483	0.376817294	-0.740404677	0.0037206
MntB	26 kDa	Membrane	0.011168563	0.919633795	-0.121038746	0.513615867	-0.522989048	0.090093231	-0.490163738	0.127930619
MntR	16 kDa	Cytoplasmic	Not detected	Not detected	-0.670841706	0.036748452	Not detected	Not detected	0.319315572	0.284172512
MogR	35 kDa	Cytoplasmic	Not detected	Not detected	-0.337638584	*	Not detected	Not detected	0.137697073	*
Mpl	57 kDa	Non-classical	2.326267906	0.008111173	Not detected	Not detected	-1.226070457	0.007555254	Not detected	Not detected
MreC	32 kDa	Membrane	-0.444554508	0.181442042	-0.286580319	0.065358541	1.481212767	0.018575325	0.290397295	0.082782262
MsrA	20 kDa	Cytoplasmic	-0.228179059	*	-0.464009688	0.191089862	0.243186571	*	0.153517754	0.625619265
MsrB	16 kDa	Cytoplasmic	-0.599530063	0.221940641	-0.405821251	0.005947129	-0.081596356	0.591607262	-0.109482046	0.389191346
MtnN	25 kDa	Cytoplasmic	-0.582360483	0.00650963	-0.378026718	0.039225173	0.77318425	0.078306645	0.015697573	0.31809795
MurA1	46 kDa	Cytoplasmic	Not detected	Not detected	-0.518401247	0.009233625	Not detected	Not detected	0.110924831	0.114647697
MurA2	45	Cytoplasmic	Not	Not	-	0.0133879	Not	Not	0.0219089	0.8183167

	kDa		detected	detected	0.429932517	73	detected	detected	11	83
MurB	32 kDa	Cytoplasmic	Not detected	Not detected	-0.115391858	0.547980113	Not detected	Not detected	0.215782255	0.082511549
MurC	50 kDa	Cytoplasmic	-0.353810427	0.326647427	-0.651873592	0.081855381	0.973292918	0.064178342	0.136263455	0.630865137
MurD	50 kDa	Cytoplasmic	-0.2694696	0.248212992	-0.435155075	0.105094218	0.451035779	0.051599879	-0.148055017	0.233921416
MurE	54 kDa	Cytoplasmic	Not detected	Not detected	-0.40038109	0.000318886	Not detected	Not detected	0.112137497	0.004987591
MurI	29 kDa	Membrane	Not detected	Not detected	-0.675591648	0.078847037	Not detected	Not detected	0.230676362	0.498012721
MutM	31 kDa	Cytoplasmic	Not detected	Not detected	-0.517198308	0.032260304	Not detected	Not detected	0.207580744	0.254834904
MutS	98 kDa	Cytoplasmic	-0.650979059	*	-0.452984137	0.16027118	0.280286571	*	0.094592549	0.574546319
MutS2	87 kDa	Cytoplasmic	Not detected	Not detected	-0.509138584	*	Not detected	Not detected	0.330897073	*
NadE	31 kDa	Cytoplasmic	-0.842307618	0.05566339	-0.445622016	0.056975428	0.547495205	0.347017448	0.044248236	0.806197741
NadK1	30 kDa	Cytoplasmic	Not detected	Not detected	-0.515624958	0.023155033	Not detected	Not detected	-0.090940605	0.195045566
NadK2	31 kDa	Cytoplasmic	-0.672260758	0.009138729	-0.717154089	0.022831494	0.962828485	0.090588064	0.082071454	0.55984487
NagB	26 kDa	Cytoplasmic	-0.473313613	0.062852658	-0.616438236	0.01083581	0.751815175	0.032500863	0.008156928	0.900626199
NamA	37 kDa	Cytoplasmic	-0.552477052	0.000550727	1.205812329	0.233087298	0.322172064	0.232307343	0.014239892	0.954766358
Ndk	16 kDa	Cytoplasmic	-0.350036087	0.067631543	-0.396004873	0.122227244	0.352540368	0.066997908	0.003949889	0.970934392
Nfo	33 kDa	Cytoplasmic	-0.645179059	Not detected	-0.395380694	0.111283915	1.009186571	*	0.097482313	0.505476911
NrdR	18 kDa	Cytoplasmic	-0.582879059	Not detected	-0.507579066	0.049483619	0.129186571	*	0.066135203	0.491946489
NusB	15 kDa	Cytoplasmic	Not detected	Not detected	-0.30784085	0.132749853	Not detected	Not detected	-0.050539764	0.828217936
Obg	47 kDa	Cytoplasmic	Not detected	Not detected	-0.4076273	0.166034341	Not detected	Not detected	-0.0614524	0.785348281

					14				77	
PanB	30 kDa	Cytoplasmic	-0.01685456	0.880802757	-0.796018103	0.11203013	-0.977174692	0.045641465	-0.196118082	0.275556359
PanC	32 kDa	Cytoplasmic	Not detected	Not detected	-0.344782786	0.179479709	Not detected	Not detected	-0.159913027	0.386839196
PdxS	32 kDa	Cytoplasmic	-0.731201561	0.194164229	-0.522828069	0.29514568	-0.071395987	0.751082986	0.004390401	0.970450287
PdxT	21 kDa	Cytoplasmic	-0.693082495	0.119514953	-0.565232299	0.036985444	0.245204284	0.286826379	0.096147637	0.667404046
PepC	51 kDa	Cytoplasmic	-0.471579059	*	-0.32135158	0.311770419	-0.309113429	*	0.212891953	0.633835892
PepT	46 kDa	Cytoplasmic	-0.45300886	0.274410825	-0.439938584	*	-0.138307294	0.51157028	0.647597073	Not detected
PfkA	34 kDa	Cytoplasmic	-0.395923426	0.056052034	-0.485171303	0.014046749	0.552317338	0.086157491	0.047535698	0.554206496
PflA	28 kDa	Cytoplasmic	Not detected	Not detected	-0.68486653	0.086144862	Not detected	Not detected	-0.031002565	0.92695726
Pgi	50 kDa	Cytoplasmic	-0.470455755	0.005854376	-0.460537161	0.011349257	0.366066904	0.00669045	0.012080699	0.803750039
Pgk	42 kDa	Cytoplasmic	-0.45946862	0.029671954	-0.597679031	0.156400496	0.74907511	0.012641444	-0.115188982	0.685135144
PheS	39 kDa	Cytoplasmic	-0.773921034	0.017564547	-0.355791423	0.042309272	0.884621037	0.015127632	0.005518263	0.971134886
PheT	88 kDa	Cytoplasmic	-1.003300201	0.059379779	-0.415370561	0.079369311	0.977992402	0.044972843	-0.180796955	0.191940994
PlcA	36 kDa	Non-classical	3.07988682	0.087670392	1.974649657	0.173634804	-1.343489971	0.151097959	-0.02592209	0.903890676
PlcB	33 kDa	Membrane	2.753185714	0.061398519	3.37273055	0.016617463	-0.703462416	0.206564167	-2.399448507	0.020448918
PlsX	36 kDa	Cytoplasmic	-0.843233158	0.045646763	-0.273469861	0.038411011	0.84554409	0.037937556	-0.103893905	0.214915833
Pnp	80 kDa	Cytoplasmic	-0.3837893	0.356195681	-0.365635369	0.019530774	0.577460933	0.251725247	0.06671504	0.526719488
PolC	163 kDa	Cytoplasmic	-1.377079059	*	-0.178231371	0.278340142	0.598286571	*	-0.122720126	0.602029101
PpaC	34 kDa	Cytoplasmic	-0.343393566	0.11348722	-0.286242427	0.229456098	0.729514643	0.041799416	-0.005233228	0.993358141

PpaX	25 kDa	Cytoplasmic	Not detected	Not detected	-0.385881137	0.076775381	Not detected	Not detected	-0.250753518	0.233475636
PrfA	27 kDa	Cytoplasmic	0.910450795	0.314223365	1.865410165	0.183990449	-0.096421278	0.478882942	-0.707415669	0.492173596
PrfB	42 kDa	Cytoplasmic	-0.123353847	0.554283012	-0.243906765	0.021246963	0.455342362	0.093637307	-0.113699378	0.186749437
PrfC	59 kDa	Cytoplasmic	0.016424156	0.973476699	-0.57862161	0.003721548	0.252999973	0.239094605	-0.039838227	0.424475528
PrmA	35 kDa	Cytoplasmic	Not detected	Not detected	-0.44621246	0.003175322	Not detected	Not detected	-0.155499452	0.309196902
PrmC	32 kDa	Cytoplasmic	Not detected	Not detected	-0.356938584	*	Not detected	Not detected	-0.205502927	*
ProB	30 kDa	Cytoplasmic	Not detected	Not detected	-0.721027411	0.198893692	Not detected	Not detected	-0.003663975	0.996412158
ProS	63 kDa	Cytoplasmic	-1.113067085	0.218700216	-0.546272994	0.294107604	0.165935791	0.662816115	0.09745586	0.839583476
Prs1	35 kDa	Cytoplasmic	-0.938818635	0.000432805	-0.856252055	0.025143189	0.80561086	0.033442545	0.053682439	0.825610931
Prs2	34 kDa	Cytoplasmic	-0.825679059	*	-0.700835809	0.068186211	0.696186571	*	0.135024998	0.609903696
PrsA2	33 kDa	Membrane	1.16876854	0.228667692	2.231417614	0.007453409	-1.935375247	0.041931123	-4.515289177	0.004573706
Pth	21 kDa	Cytoplasmic	Not detected	Not detected	-0.652368828	0.105265324	Not detected	Not detected	0.031988522	0.902997003
PtsH	9 kDa	Cytoplasmic	-0.522653809	0.291402596	-0.489281584	0.143983942	0.584712203	0.039510147	-0.066541263	0.789272645
PtsI	63 kDa	Cytoplasmic	-0.565972884	0.011774791	-0.422972546	0.044771308	0.581655202	0.037550084	0.008532427	0.949236147
PurA	48 kDa	Cytoplasmic	-0.557984126	0.060512971	-0.346287966	0.07150181	1.214496751	0.017631131	-0.020672303	0.895550425
PurC	27 kDa	Cytoplasmic	Not detected	Not detected	-0.458478058	0.047606063	Not detected	Not detected	-0.093586329	0.033658036
PurQ	25 kDa	Cytoplasmic	Not detected	Not detected	-0.412738584	*	Not detected	Not detected	-0.087202927	*
PyrG	60 kDa	Cytoplasmic	-0.330479059	Not detected	-0.685201149	0.099915617	0.809086571	*	0.200806863	0.605607938
PyrH	26	Cytoplasmic	Not	Not	-	0.1292189	Not	Not	0.1494647	0.5797137

	kDa		detected	detected	0.438657773	65	detected	detected	31	59
PyrR	21 kDa	Cytoplasmic	Not detected	Not detected	-0.727138362	3.80974E-06	Not detected	Not detected	0.262615572	0.008553452
QueA	38 kDa	Cytoplasmic	Not detected	Not detected	-0.842996073	0.078687846	Not detected	Not detected	0.009371653	0.932917979
RadA	50 kDa	Cytoplasmic	Not detected	Not detected	-0.757299258	0.004034211	Not detected	Not detected	-0.109154395	0.204693741
RbfA	13 kDa	Cytoplasmic	-0.490479059	*	-0.675082537	0.04372938	0.624886571	*	0.088341225	0.351514258
RecA	38 kDa	Cytoplasmic	-0.595046551	0.030416731	-0.26014926	0.113895018	0.061850634	0.782599343	-0.247149592	0.162784539
RecR	22 kDa	Cytoplasmic	Not detected	Not detected	-0.433734338	0.047327595	Not detected	Not detected	0.057031862	0.527326365
Rex	24 kDa	Cytoplasmic	-0.526178171	0.277449243	-0.468074824	0.027889181	1.447480123	0.065753918	0.056058737	0.677439945
RimM	20 kDa	Cytoplasmic	-0.5814845	0.027366305	-0.46375893	0.01598034	0.357623726	0.281062256	0.089297961	0.304330205
RimP	18 kDa	Cytoplasmic	-0.040179059	*	-0.363800454	0.382339435	0.420886571	*	-0.069895225	0.855730974
RlmH	18 kDa	Cytoplasmic	Not detected	Not detected	-0.548438584	*	Not detected	Not detected	0.019597073	*
RlmN	42 kDa	Cytoplasmic	Not detected	Not detected	-0.387844728	0.047236245	Not detected	Not detected	-0.074088863	0.491301706
Rnc	26 kDa	Cytoplasmic	Not detected	Not detected	-0.466135867	0.020926407	Not detected	Not detected	-0.100499807	0.275882496
RnhC	34 kDa	Cytoplasmic	Not detected	Not detected	-0.538701731	0.186900338	Not detected	Not detected	-0.11377403	0.166455913
Rnz	34 kDa	Cytoplasmic	Not detected	Not detected	-0.479938584	*	Not detected	Not detected	-0.03953626	*
Rph	27 kDa	Cytoplasmic	Not detected	Not detected	-0.536901919	0.013359993	Not detected	Not detected	0.178855965	0.297625019
RpiA	25 kDa	Cytoplasmic	Not detected	Not detected	-0.486954348	0.061896666	Not detected	Not detected	0.052978966	0.70110036
RplA	25 kDa	Cytoplasmic	-0.396135884	0.162940153	-0.525006664	0.064587144	0.855029193	0.117672135	0.10129918	0.294015675
RplB	31 kDa	Cytoplasmic	-0.4943730	0.234473729	-0.3526688	0.057516101	0.339498941	0.219675053	-0.0288590	0.599225107

			44		24				89	
RplC	23 kDa	Cytoplasmic	-0.381172619	0.161355307	-0.476763029	0.03140117	0.782128233	0.237185625	0.037723643	0.441594691
RplD	23 kDa	Cytoplasmic	-0.562789773	0.053551525	-0.429534338	0.016524974	0.444021077	0.27660352	0.068721455	0.246289441
RplE	20 kDa	Cytoplasmic	-0.042973029	0.773068323	-0.466558649	0.042912541	0.39588949	0.395625811	0.064040855	0.732390209
RplF	19 kDa	Membrane	-0.183969336	0.39912849	-0.427271303	0.049807549	0.66195054	0.240594849	0.041763814	0.018274022
RplI	16 kDa	Cytoplasmic	-0.140449871	0.863634306	0.995793412	0.169077958	-0.457768708	0.496046034	0.058698612	0.904933307
RplJ	18 kDa	Cytoplasmic	-0.478566938	0.009946193	-0.573325322	0.388674415	0.667992918	0.129804969	0.274183439	0.652186038
RplK	15 kDa	Cytoplasmic	-0.348231805	0.166919465	-0.372435867	4.21157E-05	0.354761769	0.186063356	0.112714339	0.092308175
RplL	12 kDa	Cytoplasmic	-0.063407339	0.791827742	-0.325225404	0.886939587	0.753465049	0.38414674	0.064957241	0.970720663
RplM	16 kDa	Cytoplasmic	-0.657808101	0.181824937	-0.416076837	0.019412034	0.800632589	0.178103395	0.176128041	0.127967223
RplN	13 kDa	Cytoplasmic	-0.368958578	0.371073842	-0.201987453	0.104290458	0.372551536	0.251804384	-0.043595589	0.745494878
RplO	16 kDa	Cytoplasmic	-0.222864937	0.314390053	-0.477382026	0.055853069	0.365681826	0.341543971	0.062772352	0.547582904
RplP	16 kDa	Cytoplasmic	-0.463350162	0.274839286	-0.33012148	0.204471783	0.385653067	0.227902486	-0.095925226	0.620920529
RplQ	15 kDa	Cytoplasmic	-0.662347962	0.058285117	-0.444660296	0.052721862	0.486457642	0.269969367	-0.042800012	0.090757882
RplR	13 kDa	Cytoplasmic	0.051339285	0.683368291	-0.368550376	0.058988749	0.583369184	0.022154403	-0.087183961	0.49294757
RplS	13 kDa	Cytoplasmic	-0.506023374	0.100993606	-0.450909688	0.010130832	0.421492617	0.102515791	0.043830018	0.432051802
RplT	14 kDa	Cytoplasmic	-0.464378934	0.000446982	-0.41665001	0.059572367	0.263495719	0.056594547	0.063033834	0.708960267
RplU	11 kDa	Cytoplasmic	-0.468291933	0.199385621	-0.452688894	0.077384964	0.5179516	0.068883955	0.096429504	0.26407079
RplV	13 kDa	Cytoplasmic	-0.567999108	0.365494829	-0.392185441	0.0466813	0.481835948	0.40668537	0.116651218	0.366269571

RplW	11 kDa	Cytoplasmic	-0.570856044	0.105781082	-0.447860577	0.025215581	0.675885687	0.332917787	0.115061031	0.226131319
RplX	11 kDa	Cytoplasmic	Not detected	Not detected	-0.729350576	0.112949405	Not detected	Not detected	-0.003288863	0.994828656
RplY	23 kDa	Cytoplasmic	-0.683740394	0.132482446	-0.748912951	0.010378254	-0.578664931	0.075177808	0.214571008	0.362661672
RpmA	11 kDa	Non-classical	-0.461195438	0.211207393	-0.379836896	0.106024505	0.449626813	0.031235438	-0.045449414	0.706451665
RpmB	7 kDa	Cytoplasmic	Not detected	Not detected	-0.318554915	0.63959997	Not detected	Not detected	-0.028340639	0.975432634
RpmC	7 kDa	Cytoplasmic	-0.498745391	0.252416134	-0.512078543	0.021451928	-0.14574806	0.371908268	0.099312513	0.13343202
RpmD	6 kDa	Cytoplasmic	-0.30109253	0.695430453	-0.509677472	0.020353658	1.092020114	0.121478374	0.058876623	0.70532771
RpmE2	9 kDa	Cytoplasmic	-0.386053383	0.171665625	-0.379030253	0.017451434	0.556817559	0.021383367	-0.059645254	0.022672599
RpmF1	6 kDa	Cytoplasmic	Not detected	Not detected	-0.39395841	0.378101596	Not detected	Not detected	0.199797853	0.653984953
RpmF2	7 kDa	Cytoplasmic	Not detected	Not detected	-0.390459494	0.491679635	Not detected	Not detected	0.285566829	0.46616662
RpmG1	6 kDa	Cytoplasmic	Not detected	Not detected	-0.400775085	0.428638927	Not detected	Not detected	0.071835596	0.894966879
RpmG2	6 kDa	Cytoplasmic	Not detected	Not detected	-0.337052879	0.004036845	Not detected	Not detected	-0.088658775	0.178545306
RpmI	8 kDa	Cytoplasmic	Not detected	Not detected	-0.63745631	0.057248783	Not detected	Not detected	-0.084289203	0.744635198
RpoA	35 kDa	Cytoplasmic	-0.469883917	0.031148713	-0.379264034	0.011545916	0.279531477	0.065879634	0.10595862	0.320523992
RpoB	133 kDa	Cytoplasmic	-0.578141816	0.058489429	-0.377539199	0.012283672	0.077943199	0.649266803	0.144251936	0.243642422
RpoC	135 kDa	Cytoplasmic	-0.497844593	0.291435837	-0.47077343	0.019195089	0.252956299	0.545411848	0.203613818	0.330291561
RpoE	21 kDa	Cytoplasmic	-0.626187858	0.160086555	-0.302060427	0.032560417	0.382010367	0.318543906	-0.031627674	0.774570989
RpoZ	8 kDa	Cytoplasmic	-0.467255013	0.090921475	-0.296101928	0.105995819	-0.190028562	0.380379001	0.036150512	0.350749484
RpsB	28	Cytoplasmic	-	0.0204725	-	0.0830532	-	0.8415438	-	0.3252940

	kDa		0.454907421	82	0.333770291	03	0.016905384	7	0.080683536	41
RpsC	25 kDa	Cytoplasmic	-0.525473065	0.162258422	-0.509622518	0.002411821	0.463309125	0.142182974	0.003224532	0.914194475
RpsD	23 kDa	Cytoplasmic	-0.499801112	0.009231405	-0.420178406	0.007532996	0.39466238	0.150091249	0.016718668	0.789385437
RpsE	17 kDa	Cytoplasmic	-0.325233012	0.089051148	-0.608355903	0.019119013	0.724336742	0.286510203	0.06623441	0.640028513
RpsF	12 kDa	Cytoplasmic	-0.41171153	0.158503552	-0.258024393	0.02351714	0.410781103	0.072882017	-0.178539764	0.129712892
RpsG	18 kDa	Cytoplasmic	-0.012034018	0.329300538	-0.283828862	0.121047719	0.361642939	0.314455017	0.030542195	0.696519122
RpsH	15 kDa	Cytoplasmic	-0.318817848	0.411562409	-0.461775286	0.097093527	0.876983139	0.084155999	0.102671538	0.699310874
RpsI	14 kDa	Cytoplasmic	0.04445645	0.893256596	-0.430961575	0.080754594	0.563048179	0.660299196	0.123159592	0.657910019
RpsJ	12 kDa	Cytoplasmic	-0.387639637	0.483118361	-0.403773024	0.037135656	0.595749182	0.331262067	0.089771093	0.608852177
RpsK	14 kDa	Cytoplasmic	-0.276094383	0.273037597	-0.498685326	0.058745124	0.499120467	0.271978743	-0.093322771	0.285296696
RpsL	15 kDa	Cytoplasmic	-0.606060062	0.172358167	-0.420069967	0.102961387	0.324082007	0.214317184	-0.053694941	0.409387262
RpsM	14 kDa	Cytoplasmic	-0.574959166	0.118203274	-0.475136014	0.005589084	0.472543698	0.407880551	0.140795874	0.156208023
RpsO	11 kDa	Cytoplasmic	Not detected	Not detected	-0.471252067	0.117660557	Not detected	Not detected	0.010555776	0.945324409
RpsP	10 kDa	Cytoplasmic	-0.432619544	0.166983298	-0.339977539	0.503775638	0.658999568	0.05877044	0.032803209	0.949958772
RpsQ	10 kDa	Cytoplasmic	-0.336798109	0.046309614	-0.355835721	0.100875369	0.367663984	0.261167082	-0.011888809	0.948439478
RpsR	9 kDa	Cytoplasmic	Not detected	Not detected	-0.466435	0.352845404	Not detected	Not detected	-0.012130644	0.942883521
RpsS	10 kDa	Cytoplasmic	-0.389269248	0.124145413	-0.325258251	0.17140715	0.31750667	0.128465793	-0.154550673	0.309740964
RpsT	9 kDa	Cytoplasmic	-0.39031741	0.311952483	-0.293791454	0.131257269	0.848960645	0.100581927	-0.105415484	0.315961586
RsbV	13 kDa	Cytoplasmic	-0.6638619	0.115557469	-0.2404450	0.308078866	0.88614601	0.116671921	-0.0687793	0.558003628

			55		19				18	
RsbW	17 kDa	Cytoplasmic	-0.527119253	0.061940392	-0.388671755	0.027212575	0.43560838	0.202347255	-0.182469791	0.014931323
RsgA1	33 kDa	Cytoplasmic	Not detected	Not detected	-0.325366745	0.058560909	Not detected	Not detected	-0.198395575	0.209849276
RsmA	33 kDa	Cytoplasmic	-0.698014587	0.157832938	-0.409339261	0.024270837	1.403747052	0.040884819	-0.099185456	0.382762264
RsmH	35 kDa	Cytoplasmic	Not detected	Not detected	-0.728178745	0.004457452	Not detected	Not detected	0.272494094	0.186658996
RuvA	22 kDa	Cytoplasmic	Not detected	Not detected	0.038521985	0.896073644	Not detected	Not detected	-0.296335045	0.36771397
ScpB	22 kDa	Cytoplasmic	Not detected	Not detected	-0.669638584	*	Not detected	Not detected	0.028097073	*
SecA1	95 kDa	Cytoplasmic	-0.638698642	0.13414911	-0.602701519	0.00236942	0.238156694	0.180095751	0.16441215	0.198997764
SepF	18 kDa	Cytoplasmic	-0.950250223	0.01267778	-0.527548489	0.078819051	0.545792142	0.047716823	0.399476278	0.070713228
SerC	40 kDa	Cytoplasmic	Not detected	Not detected	-0.736050552	0.039096883	Not detected	Not detected	0.344721098	0.350788517
SerS	49 kDa	Cytoplasmic	-0.512023276	0.183212791	-0.505343356	0.006492757	0.824601546	0.007234281	-0.074330061	0.398811106
SigA	42 kDa	Cytoplasmic	-0.660436661	0.017504356	-0.477244996	0.037169511	0.113381234	0.675677788	0.117434676	0.12520613
SmpB	18 kDa	Cytoplasmic	Not detected	Not detected	-0.467802855	0.010846511	Not detected	Not detected	-0.021733377	0.823931367
SodA	23 kDa	Cytoplasmic	-0.482978889	0.316865148	-0.357798524	0.094909985	0.279446769	0.504372867	0.002921567	0.976672157
SpoVG1	11 kDa	Cytoplasmic	-0.571341856	0.268901959	-0.525182793	0.012961301	0.374606824	0.152634366	0.050821528	0.636017708
SpoVG2	11 kDa	Cytoplasmic	-0.4537907	0.302965806	-0.460579394	0.005077811	-0.13828806	0.611642463	-0.226455485	0.002163105
SpxA	16 kDa	Cytoplasmic	Not detected	Not detected	-0.429810369	0.468570509	Not detected	Not detected	0.226247096	0.760855799
Ssb1	19 kDa	Cytoplasmic	-0.477064274	0.018049813	-0.255832589	0.221981521	0.647994098	0.059629362	-0.181297355	0.330973079
Stp	28 kDa	Cytoplasmic	-0.450382351	0.319822866	-0.515062582	0.03087472	0.131273088	0.673641129	0.036152972	0.223685327

Tal1	23 kDa	Cytoplasmic	-0.350706607	0.438001625	-0.483243121	0.303075186	-0.931705353	0.109345861	-0.203541381	0.692865959
Tal2	24 kDa	Cytoplasmic	-0.630770729	0.104297377	-0.60381089	0.034601188	-0.121202089	0.846660394	-0.10394023	0.889907447
TarI	27 kDa	Cytoplasmic	-0.234906505	0.245792921	-0.395333504	0.127670734	0.308360181	0.234088492	0.209703525	0.41384732
TcsA	38 kDa	Lipid-anchored	-0.730546696	0.035132008	-0.198655169	0.275450133	0.742297454	0.00635036	-0.710668152	0.003968766
Tdk	22 kDa	Cytoplasmic	-0.606352381	0.075798053	-0.532110512	0.010014147	0.428700622	0.148507145	-0.138619071	0.284823996
TetS	73 kDa	Membrane	Not detected	Not detected	-0.460183967	0.004327599	Not detected	Not detected	0.087447906	0.125015874
Tgt	43 kDa	Cytoplasmic	Not detected	Not detected	-0.265988583	0.020437769	Not detected	Not detected	0.077842579	0.325740971
ThiI	45 kDa	Cytoplasmic	Not detected	Not detected	-0.499794746	0.209990147	Not detected	Not detected	0.009179531	0.972026917
ThrB	31 kDa	Cytoplasmic	Not detected	Not detected	-0.712938584	*	Not detected	Not detected	-0.125302927	*
ThrS	73 kDa	Cytoplasmic	-0.523369787	0.014824011	-0.462288134	0.009657924	0.689868774	0.010842629	-0.053170452	0.117884668
ThyA	36 kDa	Cytoplasmic	-0.698107881	0.037395403	-0.464480204	0.025001856	0.718998563	0.221686194	0.074775652	0.047792224
Tig	48 kDa	Cytoplasmic	-1.076024169	0.099981137	-0.568234406	0.027827929	1.379763913	0.075530514	0.119676089	0.128711307
TilS	74 kDa	Cytoplasmic	Not detected	Not detected	0.560361416	*	Not detected	Not detected	-0.422802927	*
Tmk	23 kDa	Cytoplasmic	-0.364379059	*	-0.240062022	0.049996458	1.002886571	*	-0.13945147	0.23770979
TpiA1	27 kDa	Cytoplasmic	-0.647733578	0.203154384	-0.672011542	0.077188808	1.468154036	0.037651312	0.055461285	0.882308508
Tpx	18 kDa	Cytoplasmic	-0.682154641	0.112128351	-0.66614023	0.002612266	0.212187808	0.553972909	-0.036464676	0.603861816
TrhO	36 kDa	Cytoplasmic	Not detected	Not detected	-0.362938584	*	Not detected	Not detected	0.178397073	*
TrmB	25 kDa	Cytoplasmic	Not detected	Not detected	-0.3953264	0.295932221	Not detected	Not detected	-0.199175721	0.433545622
TrmD	28	Cytoplasmic	Not	Not	-	0.0251520	Not	Not	0.0229322	0.7248214

	kDa		detected	detected	0.414953557	19	detected	detected	87	52
TrmFO	48 kDa	Cytoplasmic	Not detected	Not detected	-1.048763597	0.033258361	Not detected	Not detected	0.133162135	0.692297988
TrpA	28 kDa	Cytoplasmic	Not detected	Not detected	-0.593246505	0.0452732 3	Not detected	Not detected	-0.1984807 7	0.555283896
TrpB	44 kDa	Cytoplasmic	Not detected	Not detected	-0.628738584	*	Not detected	Not detected	-0.208802927	*
TrpF	22 kDa	Cytoplasmic	-0.323979059	*	-0.333122265	0.096869281	-0.132613429	*	-0.254866881	0.171784935
TrpS	37 kDa	Cytoplasmic	Not detected	Not detected	-0.364554617	0.0786111 8	Not detected	Not detected	-0.059641148	0.688680743
TruA	28 kDa	Cytoplasmic	Not detected	Not detected	-0.543338584	*	Not detected	Not detected	0.196997073	*
TruB	34 kDa	Cytoplasmic	Not detected	Not detected	-0.450717018	0.092978503	Not detected	Not detected	0.062924788	0.661922254
TrxA	12 kDa	Cytoplasmic	-0.132988106	0.034292226	-0.444036418	0.281232759	0.249341823	0.2480940 9	0.057341327	0.865865531
TrxB	34 kDa	Cytoplasmic	-0.689670333	0.042293964	-0.2726820 7	0.032120375	0.945654972	0.011667705	0.218027048	0.011621417
TsaD	37 kDa	Cytoplasmic	Not detected	Not detected	-0.5141923 6	0.135333713	Not detected	Not detected	0.047692026	0.186366616
Tsf	33 kDa	Cytoplasmic	-0.566376027	0.0777912 9	-0.410333797	0.066866539	0.949063449	0.040293341	-0.052807785	0.704420556
Tuf	43 kDa	Cytoplasmic	-0.775190236	0.061487483	-0.455073311	0.020673095	1.439614657	0.025945097	-0.179536342	0.061169744
TyrS	48 kDa	Cytoplasmic	-0.475905477	0.0256012 1	-0.379257897	0.0828831 5	0.942210704	0.121462327	-0.060455872	0.732433658
Upp	23 kDa	Cytoplasmic	-0.6721738 8	0.027082371	-0.602268461	0.002990062	1.147800307	0.070679338	0.058670507	0.194965457
UvrA	106 kDa	Cytoplasmic	-0.714858178	0.246510023	-0.432576503	0.0153157 5	0.364776665	0.487410128	-0.0385488 1	0.267079065
UvrB	76 kDa	Cytoplasmic	Not detected	Not detected	-0.496929972	0.003283107	Not detected	Not detected	-0.081323139	0.341882782
ValS	102 kDa	Cytoplasmic	-1.037779059	*	-0.335807171	0.038693626	1.078286571	*	-0.135953292	0.214569561
Xpt	21 kDa	Cytoplasmic	Not detected	Not detected	-0.7019493	0.010427509	Not detected	Not detected	0.301665657	0.192865783

					03					
YbeY	19 kDa	Cytoplasmic	Not detected	Not detected	-0.20781532	0.334696569	Not detected	Not detected	0.129433776	0.137004664
YhaM	35 kDa	Cytoplasmic	-0.196946733	0.498387864	-0.329249353	0.126909146	-0.086720034	0.660111303	-0.007799232	0.978269297
YneA	12 kDa	Membrane	-1.144311684	0.034865318	Not detected	Not detected	-0.01823142	0.828926207	Not detected	Not detected
ZurR	16 kDa	Cytoplasmic	Not detected	Not detected	-0.229476269	0.027069699	Not detected	Not detected	0.003497732	0.895415046

* = could not be calculated

Table S6. List of oligonucleotides

Primer Name	Sequence (5'-3')	Use
prsA2-V91T	GAGATCAATTCTCTGCAacTTTAGCTCAAAGTGGC	Site-directed mutagenesis
prsA2-V91Trc	GCCACTTTGAGCTAAAgtTGCAGAGAATTGATCTC	Site-directed mutagenesis
prsA2-V28A<br>prsA2-<br>V28Arc<br>prsA2-L40A<br>prsA2-L40Arc<br>prsA2-Y41A<br>prsA2-<br>Y41Arc<br>prsA2-M44A<br>prsA2-<br>M44Arc	GTGGCGGCGGAGATGTCGcTAAGACAGACTCTGG CG<br>CGCCAGAGTCTGTCTTAgCGACATCTCCGCCGCCA C<br>GTAACAAAAGACGAAgcTTATGACGCAATGAAAG<br>CTTTCATTGCGTCATAAgcTTCGTCTTTTGTTAC<br>CAAAAGACGAAgcTgcTGACGCAATGAAAG<br>CTTTCATTGCGTCAgcAgcTTCGTCTTTTG<br>GAAgcTgcTGACGCAgcGAAAGATAAATATG<br>CATATTTATCTTTCgcTGCGTCAgcAgcTTC	Site-directed mutagenesis
LLO_SacI_F<br>LLO_XmaI_R	agtaGAGCTCAAGGATGCATCTGCATTCAATAAAGA<br>agtaCCCGGGTTATTCGATTGGATTATCTACTTTATT	Cloning

Supplementary Material References

1. Fan YX, Zhou JM, Tsou CL, Kihara H. 1998. Unfolding and refolding of dimeric creatine kinase equilibrium and kinetic studies. Protein Science 7:2631-2641.
2. Scheuermann TH, Padrick SB, Gardner KH, Brautigam CA. 2016. On the acquisition and analysis of microscale thermophoresis data. Analytical Biochemistry 496:79-93.
3. Brautigam CA. 2015. Calculations and publication-quality illustrations for analytical ultracentrifugation data, p 109-133, Methods in enzymology, vol 562. Elsevier.
4. Elfmann C, Stülke J. 2023. PAE viewer: a webserver for the interactive visualization of the predicted aligned error for multimer structure predictions and crosslinks. bioRxiv:2023.03. 06.531253.
5. Bishop DK, Hinrichs D. 1987. Adoptive transfer of immunity to Listeria monocytogenes. The influence of in vitro stimulation on lymphocyte subset requirements. Journal of immunology (Baltimore, Md: 1950) 139:2005-2009.
6. Miner MD, Port GC, Freitag NE. 2008. Functional impact of mutational activation on the Listeria monocytogenes central virulence regulator PrfA. Microbiology (Reading, England) 154:3579.
7. Alonzo F, Port GC, Cao M, Freitag NE. 2009. The posttranslocation chaperone PrsA2 contributes to multiple facets of Listeria monocytogenes pathogenesis. Infection and Immunity 77:2612-2623.

8. Cahoon LA, Freitag NE, Prehna G. 2016. A structural comparison of Listeria monocytogenes protein chaperones PrsA1 and PrsA2 reveals molecular features required for virulence. Molecular Microbiology 101:42-61.
9. Alonzo F, Xayarath B, Whisstock JC, Freitag NE. 2011. Functional analysis of the Listeria monocytogenes secretion chaperone PrsA2 and its multiple contributions to bacterial virulence. Molecular Microbiology 80:1530-1548.
